# Mathematical epidemiology in a data-rich world

**DOI:** 10.1016/j.idm.2019.12.008

**Published:** 2020-01-07

**Authors:** Julien Arino

**Affiliations:** Department of Mathematics & Data Science NEXUS, University of Manitoba, Winnipeg, Manitoba, Canada

**Keywords:** Mathematical epidemiology, Data acquisition, Open data

## Abstract

I discuss the acquisition and use of “background” data in mathematical epidemiology models, advocating a proactive approach to the incorporation of said data. I illustrate various mechanisms for acquiring data, mainly from open data sources. I also discuss incorporating this data into models.

## Introduction

Data is now the world’s most valuable resource. In 2018, the five most valuable companies in the world were, in decreasing order of valuation, Apple, Alphabet, Microsoft, Amazon and Facebook. Compare this with the situation 10 years earlier, where the top valued companies were, in decreasing order, PetroChina, Exxon, General Electric, China Mobile and ICBC.

This transition towards a data-driven world can also be apprehended when considering the wealth of information that is readily accessible on the Internet. Science is behind the technology that drives this information exchange, so it is not surprising that it would also be involved in generating some of that information. Many areas in the biological sciences are embracing this change. At the forefront, areas such as genomics and proteomics have most of their data openly accessible online. More and more ecological publications require that data be made available to others. Mathematical biology, because it is intrinsically connected to some of these domains, is also benefiting from this change. This abundance of data does not affect all areas of mathematical biology in the same way. Besides omics, population dynamics is a domain that sees a lot of information put online. However, even within population dynamics, it is important to understand that a lot of data remains difficult to access; for instance, not all epidemic propagation events see their data be made readily available.

Altogether, despite these limitations, it is becoming increasingly evident that not using data when it is available should be a thing of the past. At the very least, a modeller should be *situationally aware*. What are the orders of magnitude of the numbers of individuals in the populations under consideration? What are the time scales involved in the evolution of the quantities being studied? These lecture notes are meant to provide some initial leads on the systematic use of data in the context of mathematical epidemiology.

I have two main goals here. The first is to give a very brief overview of the abundant resources available to develop an understanding of the context in which we are operating. My second goal is to illustrate simple techniques of data incorporation in models. Note that these lecture notes barely scratch the surface of a very rich domain area. Also, other lecture notes in this special issue explain in more detail how to deal with specific problems in the use of data. My aim is less ambitious: I advocate for a more integrated use of data and present techniques that can be used to inform models with data.

This document is organised as follows. In Section 1, I discuss data sources and in particular, the distinction between proprietary and open data. In Section 2, I describe some programmatic mechanisms for accessing and acquiring open data. Even when one has data, incorporating it into models is not necessarily straightforward; in Section 3, I use the very simple example of life expectancy as a cautionary example of the issues involved. The second part of the document presents case studies related to the spatial and temporal spread of epidemics. Some data and general ideas are shown in Section 4, with further discussion on metapopulation models, a way to apprehend this type of problems. Two examples are then given; the case of an SLIAR model for the spread of a disease between 5 countries is considered in Section 5 and the same type of model is used in Section 6 to study the spread of influenza between the regions of France.

### Remarks about this document

To illustrate the philosophy of these lecture notes, this entire document is produced using Rmarkdown (link), an extension of the R programming language. Rmarkdown combines the markdown language, a simple markup language allowing LaTeX instructions and R *code chunks* that are executed when the code is run in R. I could also have used sweave, another R extension allowing, this time, to include R code in LaTeX. However, Rmarkdown has the advantage that, using almost the exact same file with very few modifications, one can also generate an html page.

The source file is accessible as an electronic appendix; it has the extension Rmd. All material is also available on my GitHub page (link). I will try to ensure that all links in this document remain current on the GitHub page; if some of the links provided here fail, refer to the document there. Data used to produce figures in this document was pulled off the web and are current as of the date of generation of the pdf of these lecture notes (2019-12-27). Some R codes are presented in the document, making for a slightly clunky feel. In a normal Rmarkdown document, this code would typically be hidden. I have hidden some instructions when they were redundant; they are nonetheless present in the Rmd file and their existence is indicated in the text by a comment. Also, in order to improve legibility, some long strings were pre-defined and comments were removed from the displayed code chunks. Finally, rather than applying a function to the result of a function, i.e., f(g(x)), I have sometimes used successive calls, i.e., x < - g(x) followed by x < - f(x).

To generate the document from the provided Rmd file, the following (free) programs are required.

• A recent version (≥3.5) of the R programming language, which can be downloaded here (link).
• Although not mandatory, using RStudio (link) greatly facilitates both R programming and, more importantly, the generation of this pdf file from the Rmarkdown source.
• A functional LaTeX installation is required.
• Several R packages (the list of packages used appears in the setup chunk of the Rmarkdown code).

Web access is also required. In order to accommodate readers with limited Internet access, the electronic appendix and GitHub repository include a copy of the data current as of the date of compilation. In the first chunk of the Rmd file, setting DOWNLOAD = FALSE will trigger the use of this downloaded data rather than online one. As a consequence, all web-based queries in the text take the form.

**Figure:**
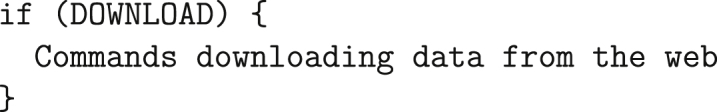

The code could be simplified by removing this check and just running said commands. Two additional remarks about using Rmarkdown to produce such a file. First, it is a good idea to name chunks, as this helps when debugging. This is easily done, by adding a name after the call to R and before any chunk options, with the chunk header taking the form.

**Figure:**
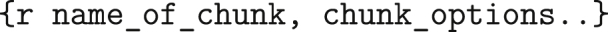

Second, in the provided Rmarkdown file, the following chunk options were set globally:

**Figure:**
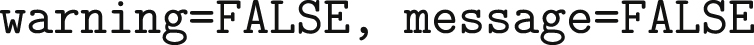

This has the effect of removing most warning messages. While this is a good idea for a production-ready document such as this one, it should be removed while developing.

## 1 Data is everywhere

### 1.1 Proprietary data versus open data

The Internet contains an enormous quantity of data, at scales that have become virtually impossible to quantify. As one searches for data on this medium, one is confronted to two main types of resources: *proprietary data* and *open data*.

Proprietary data is often generated by companies, governments or research laboratories. It is either impossible to access, or its access is heavily regulated. At the other end of the spectrum, open data is easily accessed. It is often data originating from the same sources as proprietary data, but it is released for common use, typically after a cool-off period. It is important to note that even when data is *open*, it is subject to a variety of licensing frameworks. Before using open data, it is therefore important to establish what type of licensing it is offered under; it is also important to establish citing mechanisms for that data. Another concern with open data is that it can be of varying qualities. This should be ascertained; some brief notions of data quality insurance are discussed later.

### 1.2 Open data initiatives

An exciting trend for modellers, which started 5–10 years ago and is becoming increasingly common, are open data initiatives. Such initiatives see governments (local or higher) create portals where data is centralised and made accessible, usually with very few constraints. The following illustrate these initiatives, from local to global scope:

• https://data.winnipeg.ca/
• https://open.alberta.ca/opendata
• https://open.canada.ca/en/open-data
• https://data.europa.eu/euodp/data/
• https://data.un.org/
• https://data.worldbank.org/
• https://www.who.int/gho/database/en/

## 2 Acquiring data

Before I review data acquisition methods, let me remark that even when data is readily available online, it is always good practice to keep a copy of the downloaded data once it has been acquired. Indeed, data is sometimes removed or moved, or it can be difficult to access because of poor or nonexistent internet connection or state-imposed filtering.

### 2.1 Retrieving data from open data portals

There are three main methods for retrieving data from open data portals:

1. Do it *by hand*;
2. Use the site’s API if there is one;
3. Use an R (or another language) library designed for that.

I now review these methods.

#### 2.1.1 Retrieving data ‘by hand’

This proves to be the most annoying way to acquire data from open data portals, by a large measure. To illustrate using this method, suppose I were to browse to the World Health Organisation Global Health Observatory site (link) and follow links there until I find data giving the number of reported cases of measles per country per year, for instance (link). At the time of writing, the WHO GHO data site has recently moved to a new platform, which at present allows exporting the data tables only in pdf or png form. Those interested in getting the actual data can still (at the time of writing) find this data on another part of the WHO website (link).

#### 2.1.2 Using an API

*Application programming interfaces* (API) allow *client-side* url-based access to *server-side* functions. In other words and in particular, API can be used to query databases hosted on the Internet. The presence of an API is typically indicated by long URL (web addresses) including symbols such as (?,:, *, &). API have become ubiquitous in recent years; it has become quite common to use them to perform quite a few operations server-side.

The WHO GHO data in Section 2.1.1 can be accessed using two different API. Both are well documented; the one I use below (*Athena*) is documented here (link), with, most importantly, some examples (link).

Before going into a bit more detail about the use of API, let me give the example of accessing the WHO GHO measles data mentioned in Section 2.1.1. To do so, I must construct a URL that spells out my query. In the following chunk, I list the various components required.

**Figure:**
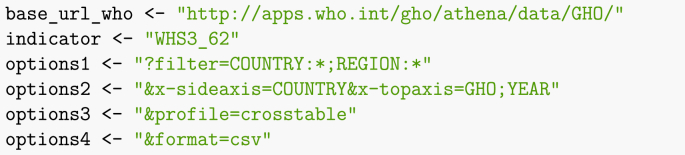

These components are relatively easy to identify, even without referring to the API documentation. options1 indicates that the result should include all countries and all regions (the different geographic groupings used by the United Nations and other agencies). options2 says the rows should be the countries/regions and columns the value of the selected index for each year. options3 specifies the type of table. Finally, options4 forces the return value to be a csv table. To get the data set in memory directly, it then suffices to reconstruct this address and use the R function read.csv.

**Figure:**
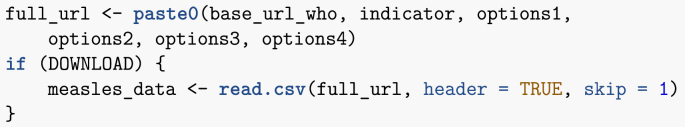

The option skip = 1 is used because there are two lines of information at the top of the csv file that are not part of the table itself. The variable base_url_who will be used again later. Using the command above, one would end up with a data frame (a common R data type), measles_data, containing the table under consideration.

Later, in Section 2.1.3, I show some examples of API for which there exists R libraries allowing easy access to the data. However, as far as I am aware, at the time there is no such library for the WHO GHO data. However, it is easy to see how the ideas that follow could be made a little more robust and turned into such a library. Therefore, let me briefly explain how one could programmatically browse the content of the WHO GHO database.

Reading the API documentation, top level WHO information comes in XML format, so I have to play with this a bit. Let me gather all the information in one place.

**Figure:**
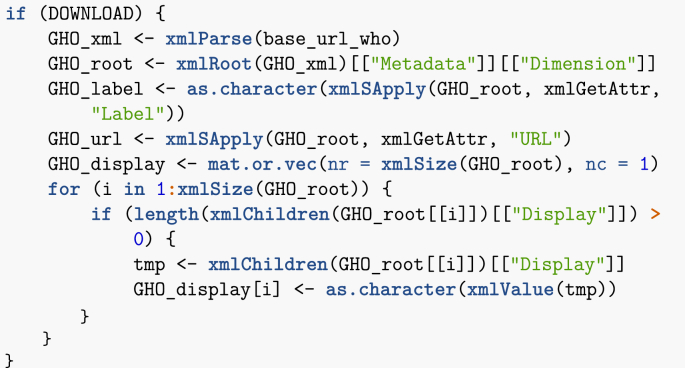

XML documents are structured documents. What the previous chunk of code does is that it browses the XML document tree. I then make a data frame of the result, then tidy up a bit.

**Figure:**
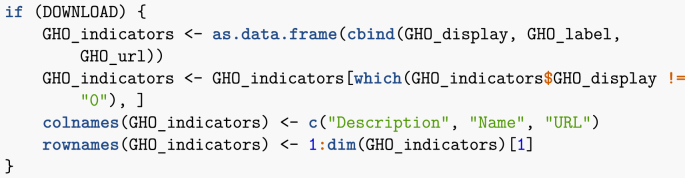

As a result of this, I have a data frame containing 3287 indicators. This data frame can now be mined for information. Let me for instance find indicators that contain the word polio. (Note that the search is performed in a case-insensitive way, so that polio and Polio are both acceptable results.)

**Figure:**
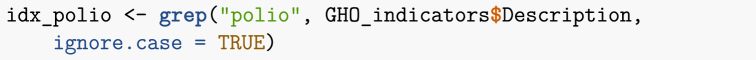

I obtain the following result.

**Table:** 

Description	Name
Poliomyelitis - number of reported cases	WHS3_49
Polio (Pol3) immunization coverage among 1-year-olds (%)	WHS4_544
Polio immunization coverage among one-year-olds (%)	poliov
Polio immunization coverage among one-year-olds (%)	vpolio

Using the name of one of the indicators in the table above (e.g., poliov) as indicator in the first chunk in this section would then allow to load the corresponding dataset.

#### 2.1.3 Using existing R librariesUsing existing R libraries

R abounds with packages allowing to perform easily the operations I carried out with the WHO GHO indicators in Section 2.1.2. (Python also has plethora of packages to access API, but for the present document I use only R solutions.) To name a few.

• WDI: query World Development Indicators (from World Bank).
• wbstats: download World Bank data; I illustrate the use of this library later.
• openstreetmap: access Openstreetmap data. This is very useful for mapping.
• tidycensus: get USA census data; this requires an API key, i.e., one needs to obtain a key from the relevant authority.
• cdcfluview: access the US CDC flu surveillance data.

Let me illustrate such libraries by using wbstats to find country life expectancy and population information.

**Figure:**
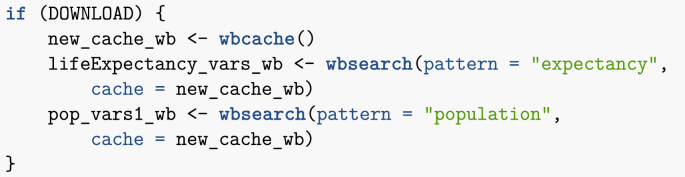

My query for life expectancy data returns 43 results. Parsing through lifeExpectancy_vars_wb, I find the index SP.DYN.LE00.IN, whose description reads *Life expectancy at birth, total (years)*. On the other hand, my search for ‘population’ returns 2464 results, so a more refined search is carried out.

**Figure:**
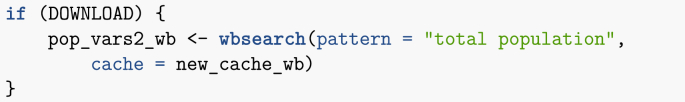

With this more refined query, I find a suitable candidate, SP.POP.TOTL, which is described as *Population, total*. I now use the function wb to download the corresponding data.

**Figure:**
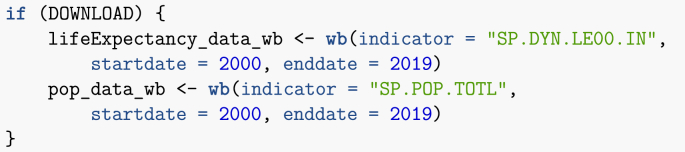

Note that the World Bank data has results for countries as well as for groups of countries. The latter are useful as they allow to work at a broader scale, both geographic (for instance, *Caribbean small states*) and economic (for instance, *Fragile and conflict affected situations*).

Since World Bank data queries are particularly important in this document, let me make a few additional remarks.

• One useful option to the function wb is mrv = , which returns the indicated number of *most recent values* for the index (or the number of values present in the database if it is lower than the number requested). See an example of usage below.
• However, not all countries have all years present. If querying for more than one country, it is best to instead give a wide range of startdate and enddate, then find the most recent year for each country, as using mrv can have an unintended consequence in this case. A function, latest_values_general, is provided in the companion file useful_functions.R, which can help with this task. See an example in Section 5.3.2.
• Using the option POSIXct = TRUE returns dates that are easier to process, especially for monthly data.

**Fig. 1:**
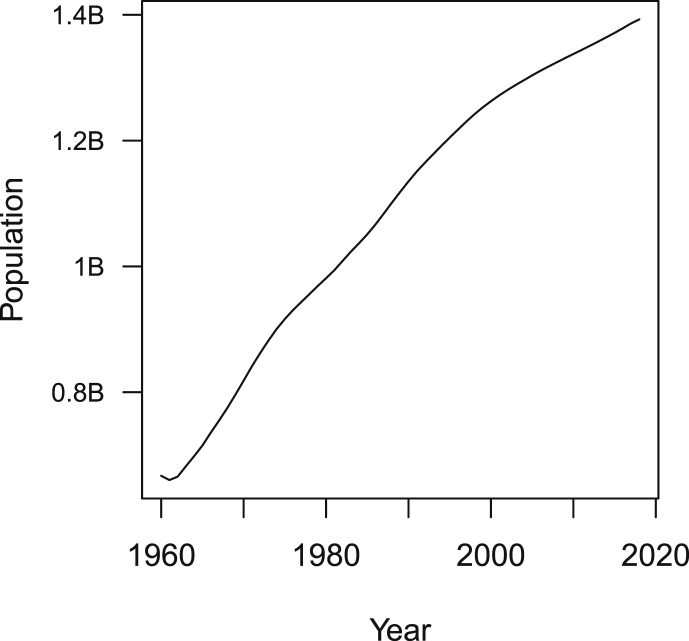
Evolution of the population of China as given by World Bank data.

Let me illustrate the use of mrv by plotting the population of China (Fig. 1). Remark that it is often useful, when exploring data or presenting simulation results, to ensure that axes are easy to read. So instead of the usual plot command, I use the function plot_hr_yaxis (in the file useful_functions.R) that labels units of the *y*-axis of the plot in a more human readable way.

**Figure:**
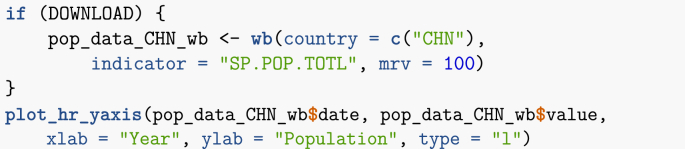

### 2.2 Something intermediate – htmltab

While being able to obtain data by querying an API is ideal, there are many cases where this is not an available option. It is however easy to grab data from tables found on web pages, using the R library htmltab. Remark that it is also possible to extract data from tables in pdf files, but this is not covered in these notes. To illustrate the use of htmltab, let me compute the population density of countries using two tables grabbed from Wikipedia (link1,link2). Note that this is a futile exercise, as Wikipedia also includes tables with population density information, but it serves to illustrate another important R command, which allows to merge tables.

Be careful when compiling this Rmd file: Wikipedia comments can appear as tables, so you may have to change the option which = 2 as it is currently set to a different value if the following commands yield an error.

**Figure:**
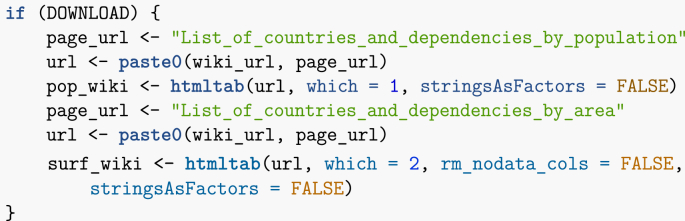

Now that the data has been acquired, some processing is needed. Tables in Wikipedia show numbers with comma separating groups of three digits; these commas need to be removed. Surface areas are also provided in both kilometres and miles; I remove the latter. Finally, I rename some columns for convenience.

**Figure:**
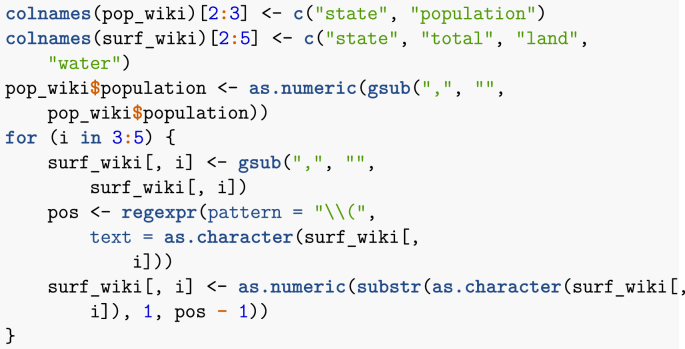

Note that htmltab allows to apply a function to each column in the table, so some of this processing could have been carried out while the table was being downloaded. I now use an important function, merge, to merge data frames with columns containing some common entries. This performs the equivalent of a JOIN command in SQL.

**Figure:**
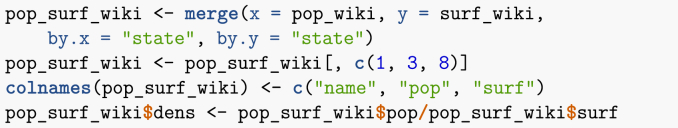

Let me now show how to map spatial results. There are many R libraries for mapping. One possible way is to proceed as follows. First, I need to translate place names into ISO 3166 (country) codes, set up bins for values and set up a colour palette. Note that bins are set up here using 20 percentiles. Indeed, using a linear scale results in too little contrast.

**Figure:**
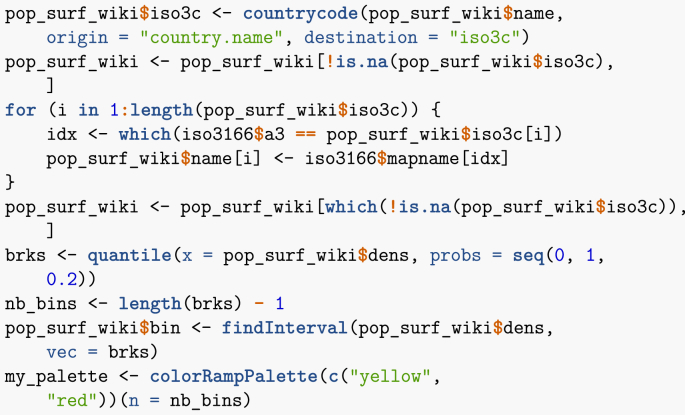

**Fig. 2:**
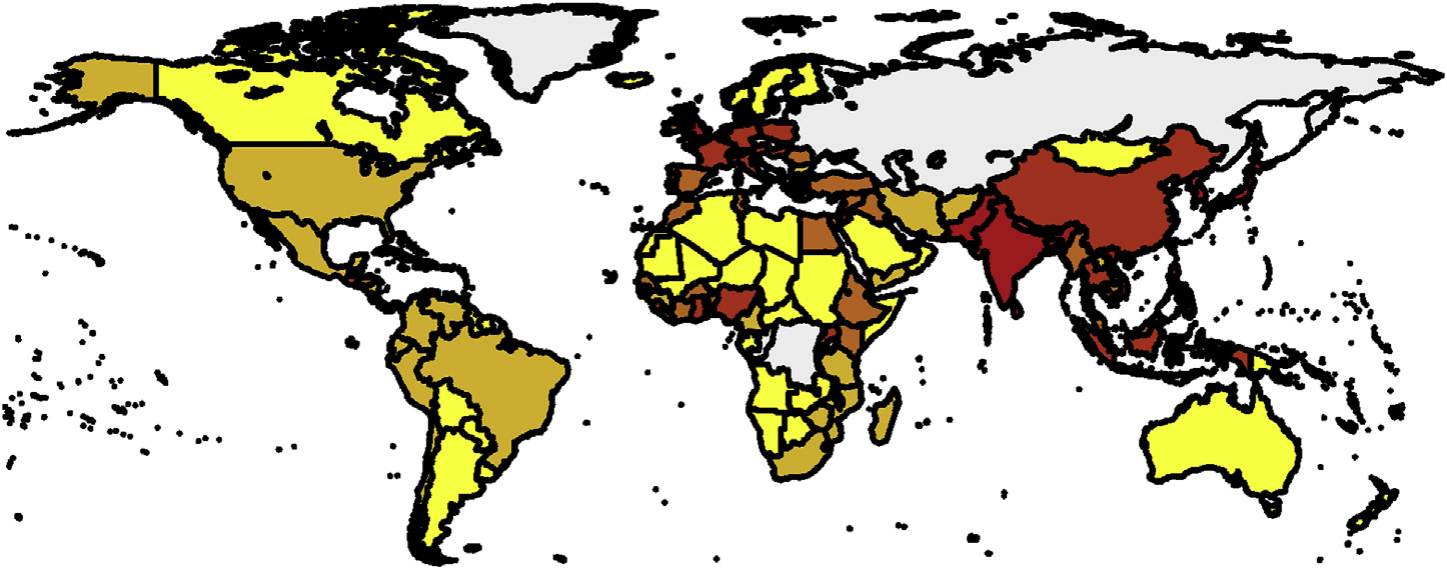
Population density.

Note that I have pruned the list of countries to get rid of ones that pose problem to the mapping routine (typically, small overseas dependencies). Proper use would dictate to go through the list of errors and establish the corresponding region name. Now that places have been binned in terms of their 20 percentile, I plot them (Fig. 2).

**Figure:**
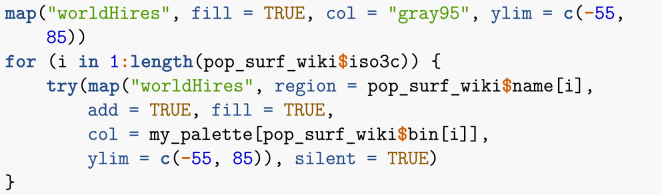

### 2.3 The extreme – data extraction from figures

One rather anecdotal method is available when data is not open *per se*, which is to capture numerical data from a figure in a publication. Note that this is by far the most time onerous method of data acquisition and should typically be a last resort option.

As soon as work is published, the figures contained therein indeed become part of the common good and the data there can be used, with proper citation of the original work. Many publications nowadays encourage publication of the data, so one should first check if the data used in the paper has been published. In the case where the data is not available online, though, for instance in old papers, one can digitise using programs such as Engauge Digitizer (link) or g3data (link). These programs typically present an interface in which the figure whose data must be digitised is displayed. In a first step, the user enters several reference points on the figure with known positions, for example, the origin and two points known on coordinate axes. Then each point of the data is clicked on and the result is generated as a csv file.

To illustrate this method, let me first download the US census data from Wikipedia and plot it. In this case, I save the figure to a file in order to then process it through the digitiser. Note that to make the plot easier to use, I also print a grid, which makes setting reference points easy.

**Figure:**
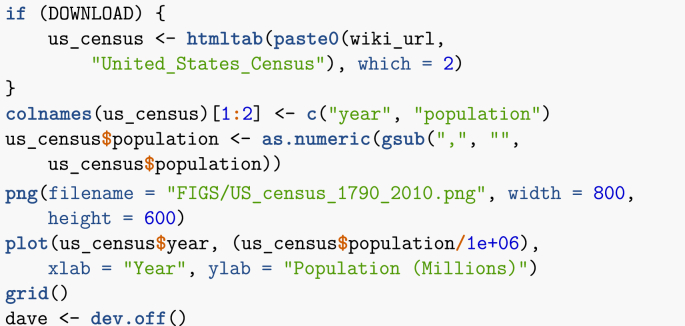

Note that the last instruction is used to completely remove the output of using dev.off(), which, interestingly, does not seem to obey the warning = FALSE, message = FALSE directive given to knitr.

**Fig. 3:**
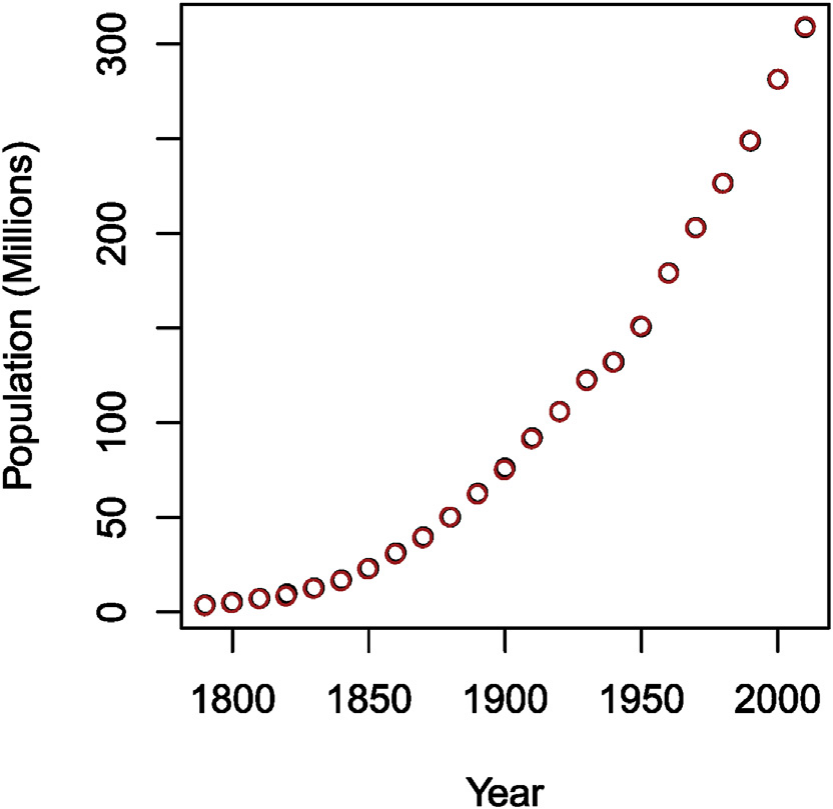
Number of people in the USA as given by the census. Black: original data; red: digitised data.

After processing this image with Engauge Digitizer, I obtain a csv file (provided in the electronic appendix). In order to visually check the result, I plot in Fig. 3 the content of this file (in red) together with the original data (in black).

Clearly, the agreement is very good, since one can barely see any black, meaning the red points completely cover the black ones. The situation was made as ideal as possible, however. In practical cases, the agreement might not be as good. This should be considered a last resort method and good practice dictates that additionally to citing the work from which the data originates, one should additionally mention that the data is digitised, since errors stemming from imprecision in digitisation should not be blamed on the authors of the original work.

## 3 Incorporating data in models – initial remarks

Now that the most basic mechanisms for acquiring data from the web have been explained, let me discuss how this data can be incorporated into population dynamics models. Remark, however, that while it is important to be aware of the magnitudes and time scales involved in the processes studied, it is also important to not use data just for the sake of using data. Also, note that I do not discuss in these lecture notes another aspect of data acquisition that is very important, namely the verification and validation of data.

There are a variety of ways for data to be incorporated into dynamical models. The main are as parameters or initial conditions, or as time series with which model solutions can be compared in order to identify other parameters. In essence, as far as model simulations go, parameters and initial conditions can be thought of as being of the same nature, mimicking the theoretic similarity between continuous dependence on initial values and parameters. So, by abuse of language, in these notes, I often call parameters both parameters and initial conditions. Parameters can be classified for instance in terms of their reliability or the way they are derived.

1. *Reliably known* parameters are related to geography, population, vaccination coverage, region centroids, etc. A typical example would be the population of a country; there can be some uncertainty about the exact value, but the *posted* value can be taken as given.
2. *Known* (or relatively well known) parameters are disease characteristics such as the incubation period or duration of the infectious period. These are typically derived from expert knowledge based on statistical analysis of characteristics.
3. *Imputed* parameters are parameters whose values are not known precisely but can be computed from known parameters by making some assumptions on processes. A typical example is the vaccination rate: it can be derived in a given model from the knowledge of the vaccine coverage.
4. *Identified* parameters are typically obtained by comparing the outcome of the model with a known time series given as data.

### 3.1 Example – Life expectancy

To illustrate the difficulty of dealing with data, let me consider what is, at first glance, a very easy parameter: *life expectancy*. Suppose I am tracking a cohort of individuals born at a certain time $t_{0}$, where the only cause of death is natural death, which occurs at the *per capita* rate *d*. (Because I am tracking a cohort, there is also no birth into the population.) Without going into details (see for example (Thieme, 2003)), the hypothesis underlying the differential equation for the rate of change of the number N(t) of individuals in this population,

$$N^{,}=-dN,$$

is that the duration of life for each individual is exponentially distributed with mean 1/d. The appropriateness of this hypothesis depends on the aim of the model.

• If one uses the model for long term predictions, then the hypothesis is valid, since over several generations, the important characteristic can be safely assumed to be the mean duration of life. In this case, we can just set 1/d to be the life expectancy data grabbed earlier.
• Now suppose that the model is to be used for short term predictions. In this case, the hypothesis of exponential distribution of life durations becomes a problem, as I illustrate in the following example.

**Fig. 4:**
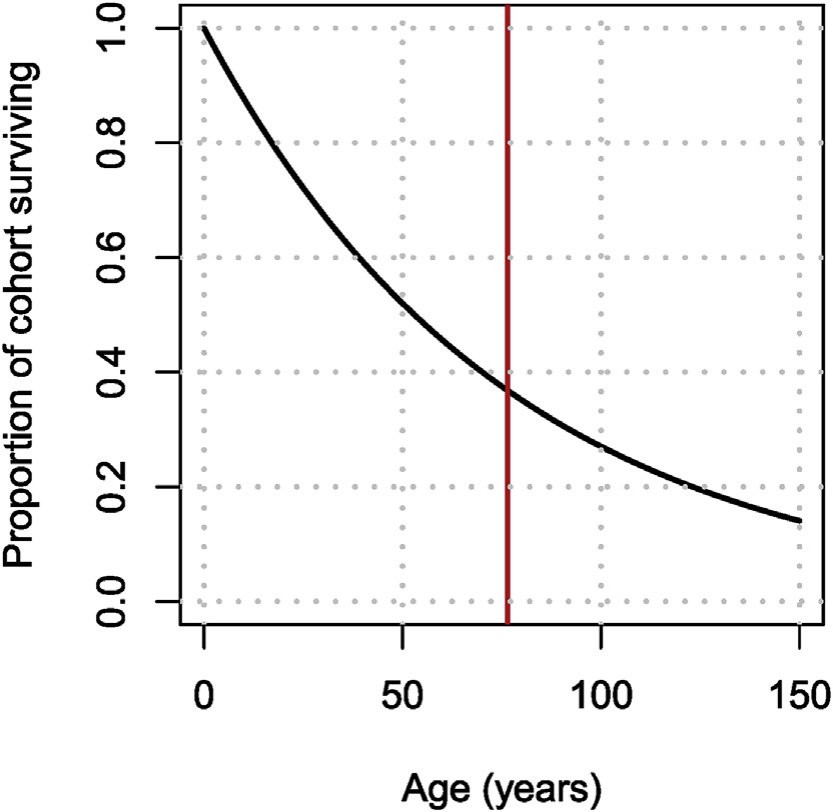
Proportion of a cohort surviving when the mean of the exponential distribution equals the life expectancy at birth in China in 2017.

Let me consider the population of China. Querying the World Bank data, life expectancy at birth in China in 2017 was 76.47 years. Now recall that for a random variable with exponential distribution with parameter *d* (or mean 1/d), the survival probability is given by $S\left(s\right)=P\left(t>s\right)=1-P\left(t\leq s\right)=e^{-ds}$. Using the value 76.47 years for the inverse of the death rate, it follows that the proportion of individuals in a cohort born at time $t_{0}=0$ who survive to age t=s is as shown in Fig. 4.

If considering a model for the long term behaviour of the population, the important characteristic is the red vertical line, i.e., the mean. However, if one is interested in the short term dynamics, then there are two issues:

1. The initial attrition is too high. In Fig. 4, only 80% of the initial cohort remains after 30 years, which is much less than what it is in real life.
2. The tapering off is too slow. Almost 20% of the cohort survives to be 150 years old.

Here, I show two simple methods for addressing this problem. There are many others, more appropriate (but more complex) ones.

• Refine the parameter of the exponential distribution to take into account known survival to given ages.
• Use the fact that the Erlang (Gamma) distribution is the sum of exponential distributions, i.e., add compartments.

#### 3.1.1 Refining the parameter of the exponential distribution

Looking through World Bank indicators as in Section 2.1.3, I find the indicator SP.POP.65UP.TO.ZS that has “Population ages 65 and above (% of total)”, which I could use to refine the survival function. Call $p_{65}$ that proportion, then

$$S\left(65\right)=p_{65}\Leftrightarrow e^{-65d}=p_{65}\Leftrightarrow d=-\left(\ln p_{65}\right)/65.$$

Grabbing the data from the World Bank, 10.92% of the population of China was over 65 in 2018. From the formula above, in order to have the (exponentially distributed) lifetime such that $S\left(65\right)=p_{65}$, the mean lifetime should be 29.35 years.

**Fig. 5:**
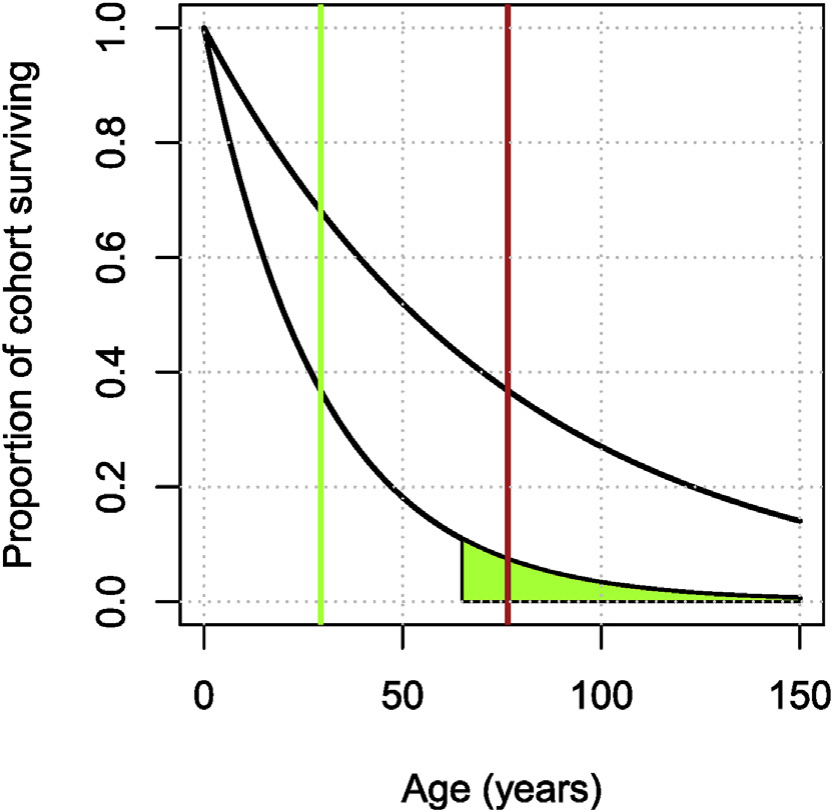
Adjusted (exponential) life expectancy distribution for CHN, taking into account the desired area under curve for survival of 65 years and older.

Fig. 5 shows the original and the adjusted distributions. Note that while the slow tapering off is resolved with this new value of *d*, this comes at the price of an even higher early attrition of the population.

#### 3.1.2 Using an Erlang as a sum of exponentials

Let $X_{i}$ be independent exponentially distributed random variables with parameter ξ and $Y=\sum_{i=1}^{n}X_{i}$. Then, the random variable Y⇝E(n,ξ), an Erlang distribution with *n* the *shape* parameter and ξ the *scale* parameter. (An Erlang distribution is a Gamma distribution with integer scale parameter.)

**Fig. 6:**
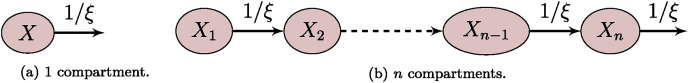
(a) Single compartment case, the time of sojourn is exponentially distributed. (b) Multiple compartments case, the resulting time of sojourn in the chain of compartments is Erlang distributed.

**Fig. 7:**
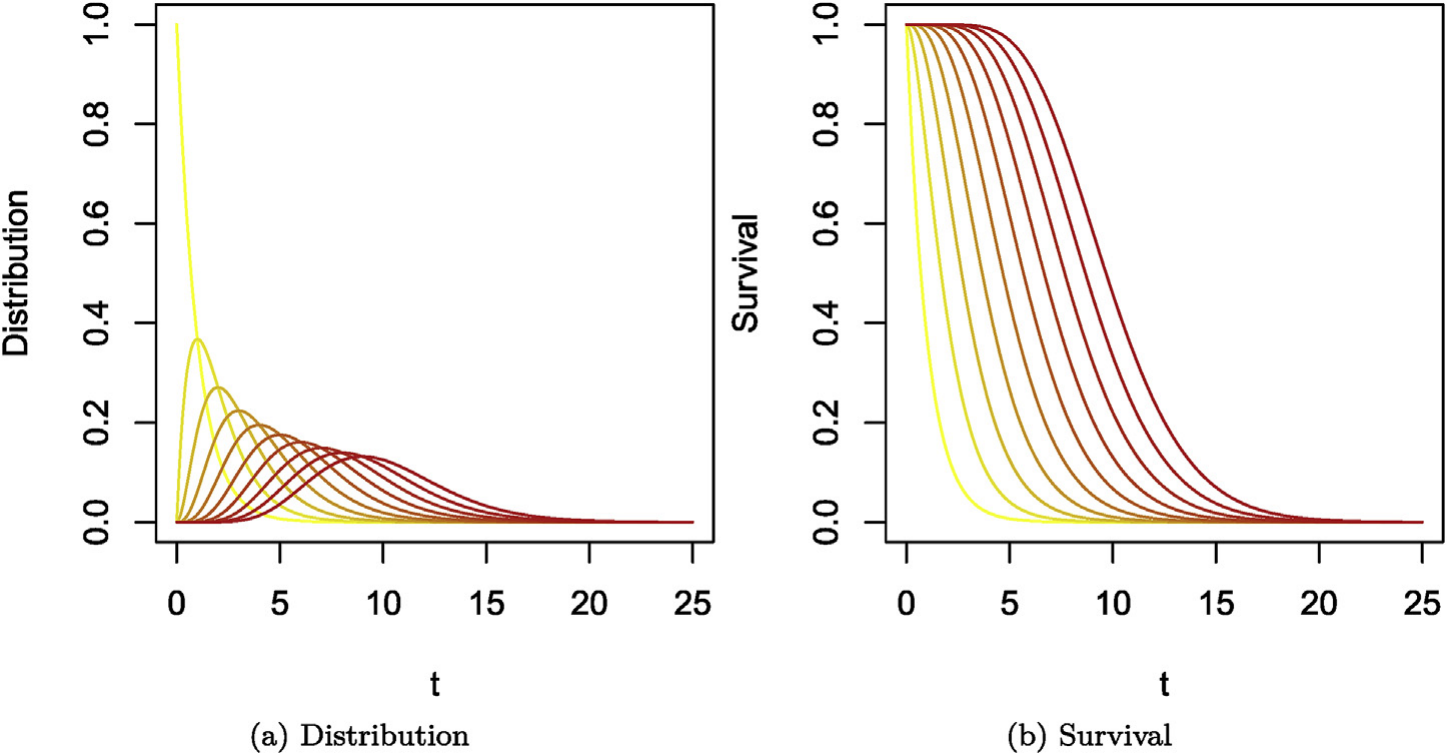
Erlang distributions with rate equal to 1 and shape parameters varying from 1 (yellow) to 10 (red). (a) Distribution. (b) Corresponding survival.

In terms of compartmental models, this means that if *n* compartments are traversed successively by individuals, with each compartment having an outflow rate of 1/ξ (or a mean sojourn time of ξ), then the time of sojourn from entry into the first compartment to exit from the last is Erlang distributed with mean E(Y)=nξ and variance $Var\left(Y\right)=n\xi^{2}$. This is illustrated in Fig. 6. For a single compartment as in Fig. 6a, the time of sojourn is exponentially distributed following the left-most (yellow) curve in Fig. 7. Adding compartments with the same mean sojourn time per compartment results in increasingly red curves in Fig. 7. Fig. 7 shows the corresponding survivals.

As an example of the use of adding compartments to fit known sojourn time distributions, let me consider the incubation period for Ebola Virus Disease. During the 2014 EVD crisis in Western Africa, the WHO Ebola Response Team estimated incubation periods in the paper (WHO Ebola Response Team, 2015). Table S2 in the Supplementary Information in (WHO Ebola Response Team, 2015) gives the best fit for the distribution of incubation periods for EVD as a Gamma distribution with mean 10.3 days and standard deviation 8.2, i.e., nε=10.3 and $\varepsilon \sqrt{n}=8.2$. From this, I obtain that $\varepsilon =8.2^{2}/10.3≃6.53$ and $n=10.3^{2}/8.2^{2}≃1.57$. However, that is a Gamma distribution.

In order to fit within the context of using multiple compartments to better fit residence times, since the number of compartments is an integer I need to find the closest possible Erlang distribution to this Gamma distribution. To do this, let me compute the square of errors between data points generated from the given Gamma distribution and an Erlang. The following function computes the square of the difference between data points $\left(t_{i},d_{i}\right)$ and a Gamma distribution with shape shape and scale theta, evaluated at the same $t_{i}$. (To get an Erlang distribution, shape needs to be an integer.)

**Figure:**
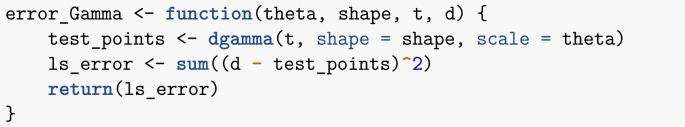

The following function takes as input data points $\left(t_{i},d_{i}\right)$ and finds optimal scale and integer shape parameters (so an Erlang distribution) corresponding to these data points. Note that the shape parameter is (arbitrarily) limited to 10, i.e., I allow at most 10 compartments. Note that I use try to avoid issues linked to the potential non-success of the call to optim.

**Figure:**
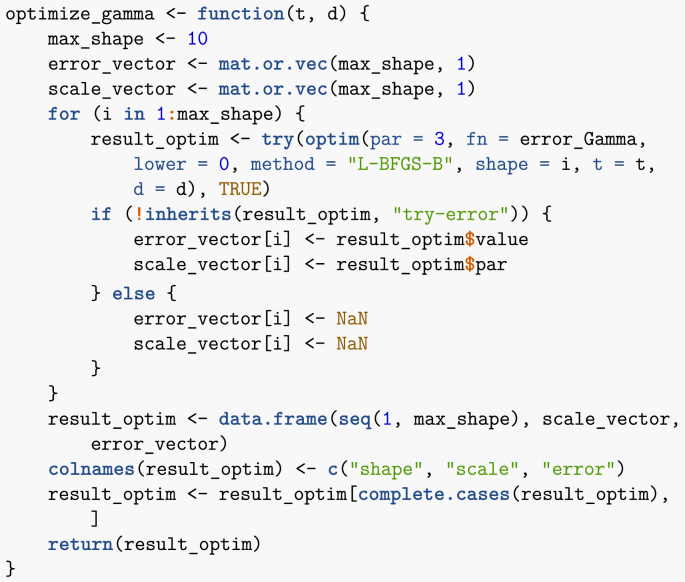

Finally, I call the function above with parameters of the Gamma distribution as given in the paper. If you had your own data points, you could use them instead in the chunk below. (The points in time for your data would be in the vector time_points, while the corresponding values would be in data_points.)

**Figure:**
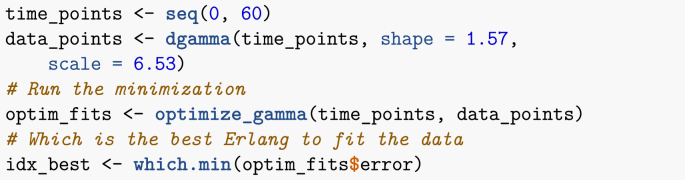

**Fig. 8:**
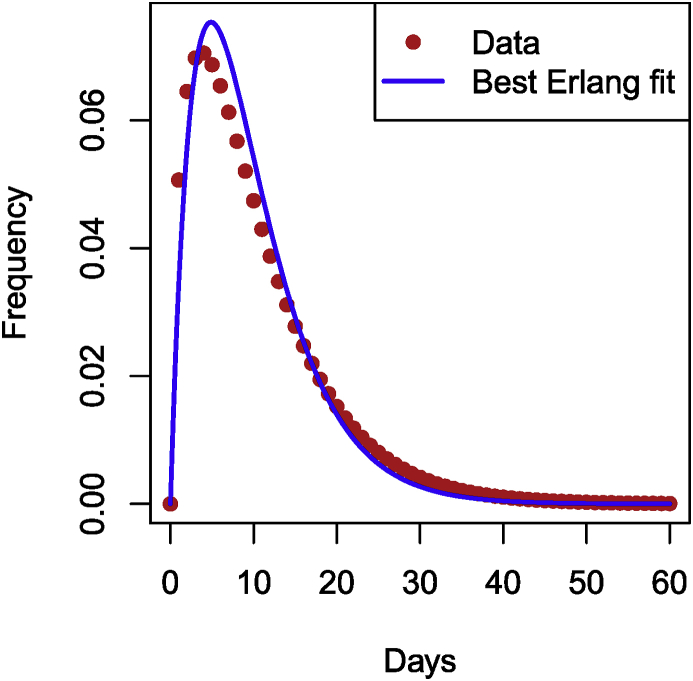
Best Erlang fit of the Gamma given in (WHO Ebola Response Team, 2015).

Now plot the result as well as the original curve, giving Fig. 8 (code chunk not shown).

## 4 Spatial spread of epidemics of humans

I now consider two real-life examples dealing with the spatial spread of epidemics, with focus on epidemics of human diseases and more specifically, influenza. I first motivate the need to consider the spatial spread of infections, using some of the techniques presented in the preceding sections of these lecture notes.

### 4.1 Motivation – pathogen spread has evolved with human mobility

Pathogens infecting humans spread over space and time together with the humans carrying them. As a working definition, let me define *mobility* as the collection of processes through which individuals change their current location. Until the advent of leisure travel in the early 20th century, long range human mobility was mainly along trade routes. Now mobility has evolved and as a consequence, so has the spread of pathogens humans carry.

Using the very broad definition of mobility given earlier, it is clear that the scale of modern mobility is difficult to apprehend. It takes many different forms, is constantly evolving and involves numbers that are colossal.

In order to illustrate one of my points in these lecture notes, namely, that a modeller needs to be situation-aware, let me show how one could gather evidence concerning the evolution of travel. This evidence will not be used directly in the models, but I do feel that its knowledge is important in model formulation and simulation.

**Fig. 9:**
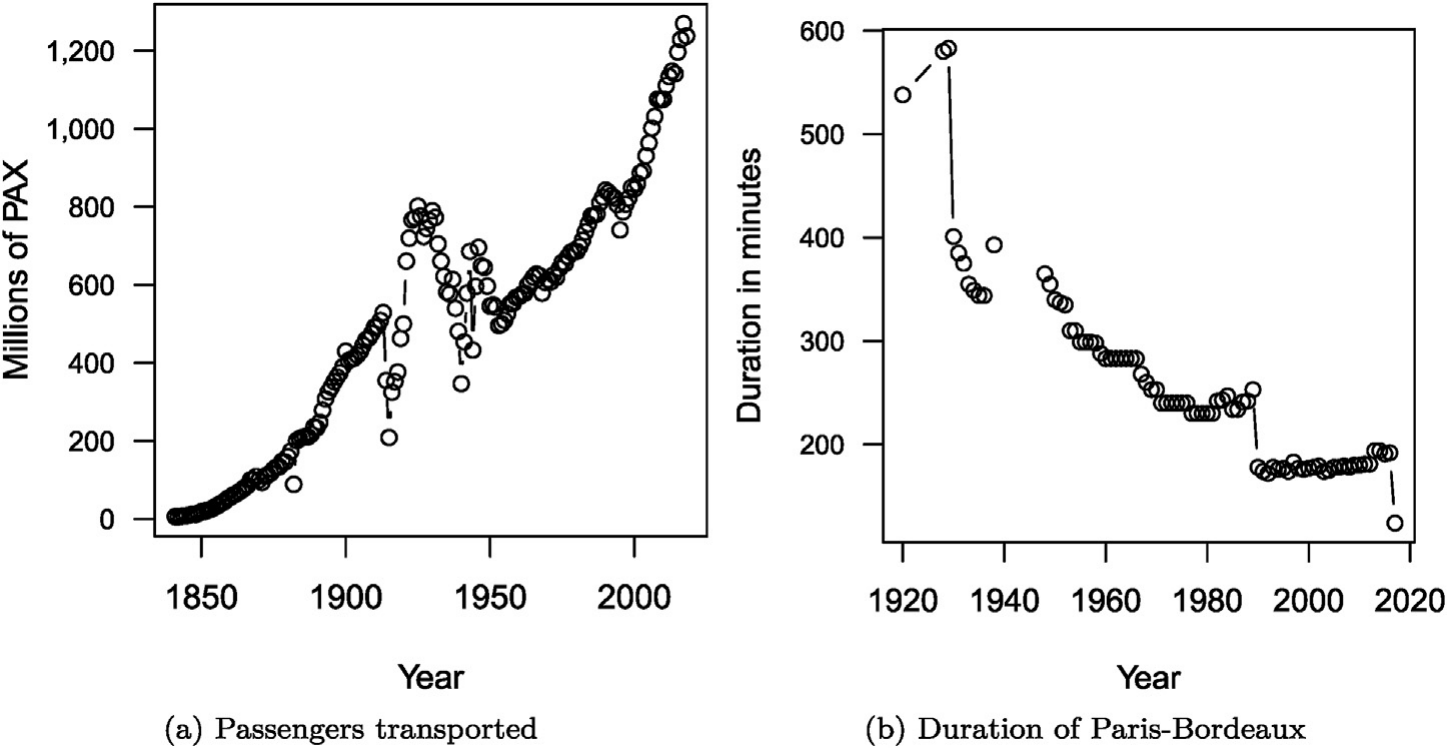
Evolution of the annual number of passengers transported since 1841 and of the duration of a Paris to Bordeaux trip since 1920, by train, in France.

In Fig. 9, I show the number (in millions) of people-trips taken on the French national railway network (SNCF) since 1841 and the evolution of the duration of a trip between Paris and Bordeaux since 1920 on the same network. (Code chunk not shown.)

**Fig. 10:**
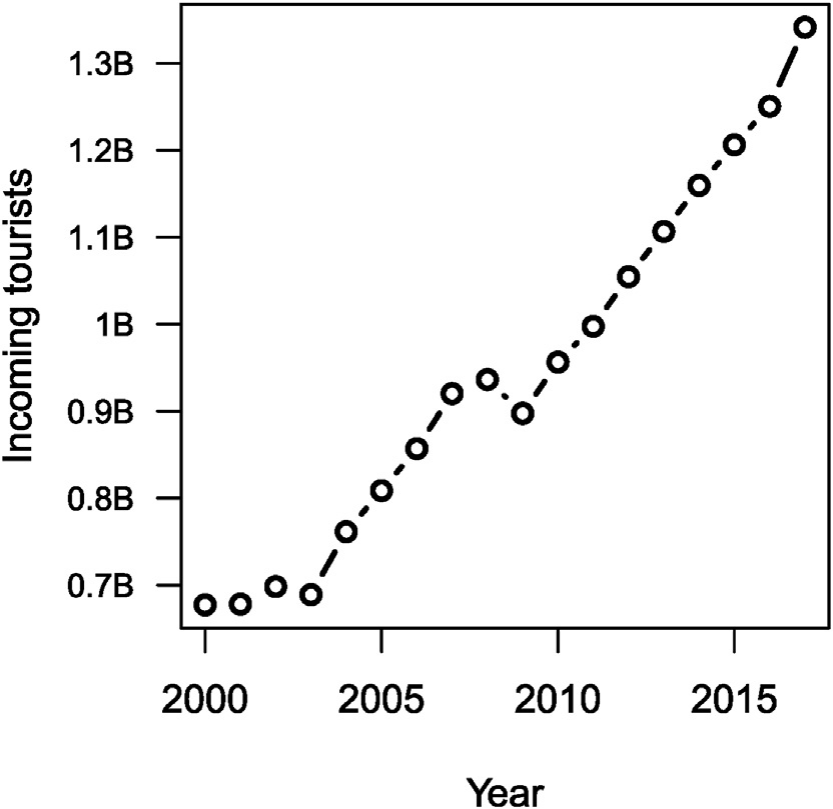
Evolution of the number of incoming tourists worldwide.

Thus, clearly, the number of passengers transported by train has increased considerably over the past 150 years, while the amount of time it takes for passengers to cover distances has dropped. Another interesting component is the number of incoming tourists worldwide, obtained from the World Bank. This is shown in Fig. 10.

Note that in the chunk used to obtain this figure, I illustrate another method for processing data: the use of the sqldf library, which allows to use SQL-type queries on R dataframes. Note that WLD stands for World.

**Figure:**
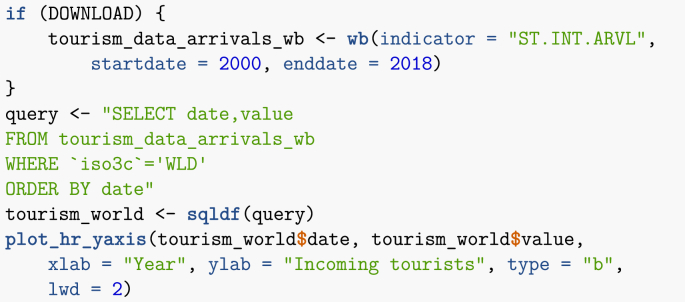

Another increasingly popular method for parsing through data not illustrated in these lecture notes are the libraries in the tidyverse. They are extremely useful, but as someone who has used SQL a lot in other context, I tend to use sqldf more. If you are agnostic in terms of method, I do recommend learning the tidyverse.

**Fig. 11:**
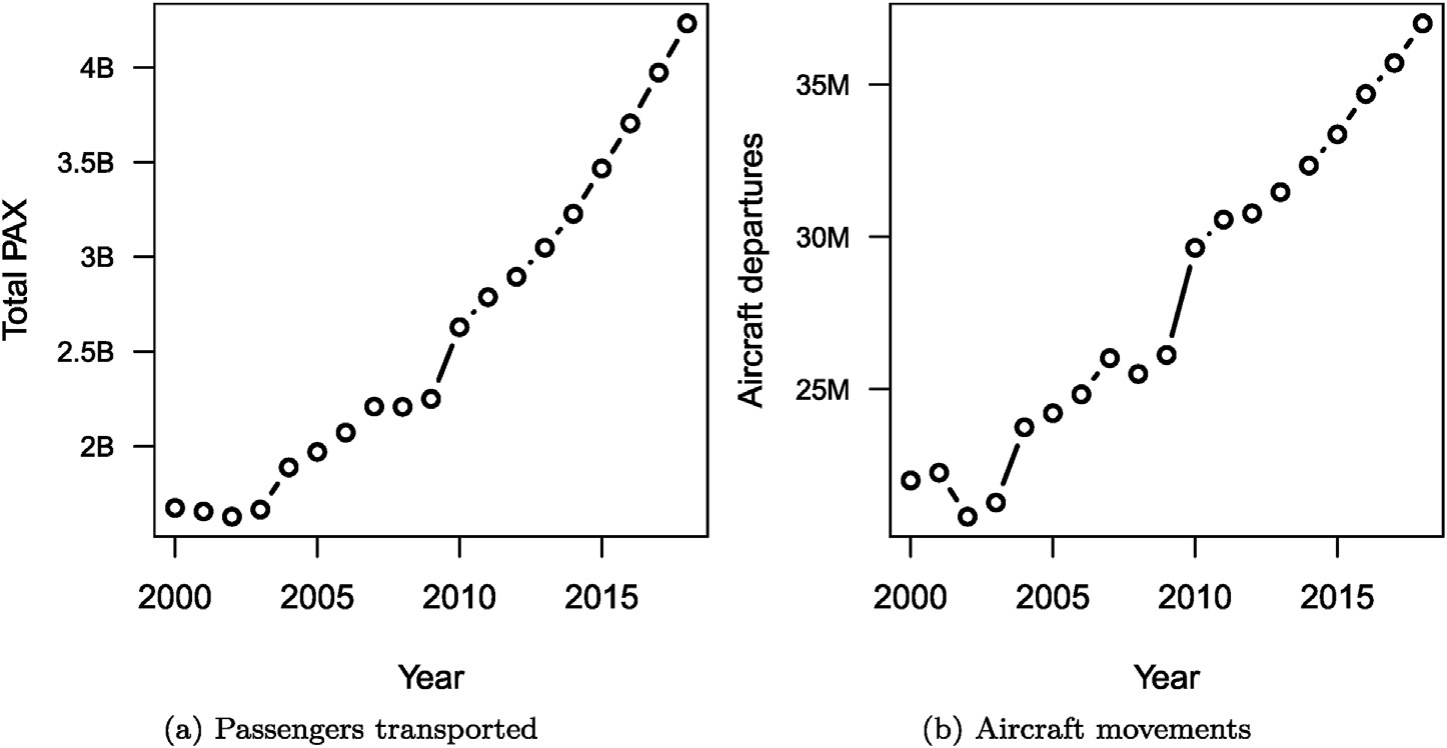
Evolution of the annual number of passengers transported by air transport and of the number of aircraft movements.

The massive increase (almost doubling) in inbound tourists since the early 21st century is due to a large extent to the increase of tourism from China and other emerging markets. A lot of tourism travel involves air travel and the rise of numbers in this context is also quite visible, as shown in Fig. 11, with data also originating from the World Bank. (Code chunk not shown.)

As a final illustration of the importance of mobility, this time at a more local scale, see this animation of the scheduled positions of buses in the City of Winnipeg (link). To create the animation there, the bus schedules were downloaded and plotted. (The code is available on the linked page.)

### 4.2 Metapopulation models

Since mobility has become such an important component of the every day life of humans, one needs ways to model this in relation to the spread of infectious diseases. There are many different approaches to do this. One of them is using so-called *metapopulation* models.

I give here a very short introduction to the topic. Refer to other work on the subject for a more detailed presentation; for instance, see (Arino, 2017) and the references therein.

#### 4.2.1 Quick introduction to metapopulation models

Metapopulations split space into |P| geographical locations called *patches* and are thus appropriate for the description of phenomena involving discrete regions rather than continuous space. Each patch contains *compartments*, relatively homogeneous groups of individuals, e.g., susceptible humans, infected humans, etc. Individuals in a compartment *may* move between patches; $m_{cqp}$ is rate of movement of individuals from compartment c∈C from patch p∈P to patch q∈P. Each patch is equipped with a system describing the evolution of the number of individuals in each compartment present. For epidemic models, the general form is as follows. Assume *uninfected* (*s*) and *infected* (*i*) compartments in sets U and I, respectively, with U∪I=C. For all k∈U, ℓ∈I and p∈P,

(1a) $$s_{kp}^{’}=f_{kp}\left(S_{p},I_{p}\right)+\sum_{q\in P}m_{kpq}s_{kq}$$

(1b) $$i_{\ell p}^{’}=g_{\ell p}\left(S_{p},I_{p}\right)+\sum_{q\in P}m_{\ell pq}i_{\ell q},$$

where $S_{p}=\left(s_{1p},\ldots ,s_{\left|U\right|p}\right)$ and $I_{p}=\left(i_{1p},\ldots ,i_{\left|I\right|p}\right)$ are the discrete distributions of individuals in the different compartments in patch p∈P. The functions *f* and *g* describe the interactions between compartments in a given patch, while the sums describe the movement of compartments between locations and are written compactly by assuming that

(2) $$m_{cpp}=-\underset{q\in P}{\sum }m_{cqp},\;\forall c\in U\cup I,$$

i.e., by denoting $m_{cpp}$ the rate of movement out of patch p∈P for individuals from compartment c∈U∪I.

## 5 An SLIAR model for five countries

Let me consider the example of influenza, for which a lot of data is available online. A basic model for influenza is the SLIAR model (Arino, Brauer, van den Driessche, Watmough, & Wu, 2006).

Before proceeding further, remark that it is important to understand why a model is being used, as this determines the nature of the modelling framework used (ODE, PDE, Markov chains) and the type of model that is formulated. Here, I want to provide a reasonably realistic simulation context in which the spread of influenza between countries or regions in a country is modelled over *one* season. The spatial context calls for a metapopulation framework, while modelling influenza over one season means that an SLIAR model without demography is appropriate.

### 5.1 Base model in each patch – SLIAR without demography

**Fig. 12:**
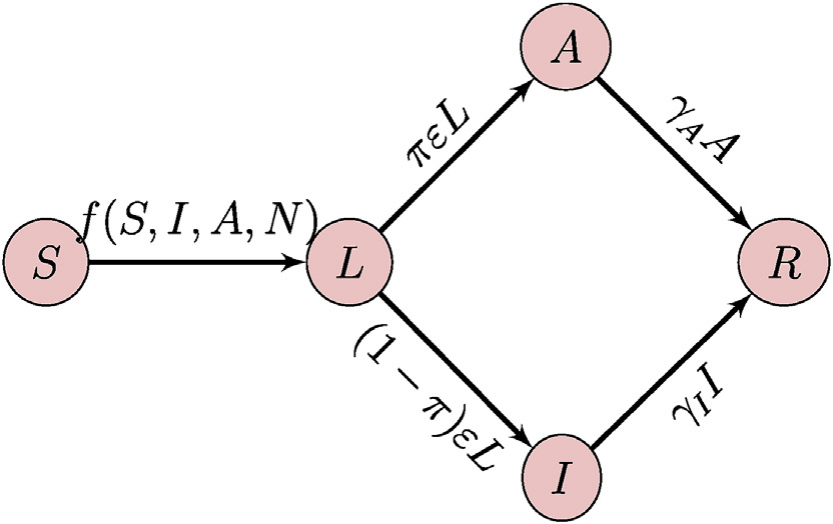
Flow diagram of the SLIAR model used in each patch.

When formulating a metapopulation model, it is good to start by clearly establishing the model that is used in each patch. Here, the population in each patch is divided into five compartments as a function of the epidemic status of individuals. Susceptible individuals (S) might be infected by the disease; upon infection, individuals go into a phase where they are incubating with the disease (L); after the incubation period finishes, individuals can become either symptomatically (I) or asymptomatically (A) infectious to others. Finally, after recovery, individuals are immune to reinfection with the strain of influenza they were infected with (R). In terms of the notation of (1), here U={S,R} and I={L,I,A}. The flow diagram of the model is shown in Fig. 12.

Parameters are as follows: infection occurs at the rate f(S,I,A,N), where N=S+L+I+A+R; the incubation period lasts on average 1/ε time units; the proportion of individuals becoming asymptomatically infectious is π; finally, the infectious period lasts on average $1/\gamma_{I}$ and $1/\gamma_{A}$ time units for symptomatically and asymptomatically infectious individuals, respectively. I do not consider disease-induced death here. As a consequence, the SLIAR model takes the form

(3a) S’=−f(S,I,A,N)

(3b) L’=f(S,I,A,N)−εL

(3c) $$I^{’}=\left(1-\pi \right)\varepsilon L-\gamma_{I}I$$

(3d) $$A^{’}=\pi \varepsilon L-\gamma_{A}A$$

(3e) $$R^{’}=\gamma_{I}I+\gamma_{A}A.$$

Note that at present, the form of the incidence function *f* has not been specified. What form to use depends on the aim of the model (McCallum, Barlow, & Hone, 2001). In the following, η≥0 is a multiplicative factor indicating the change in infectiousness due to being an asymptomatic case. Typically, it is assumed to be in [0,1].

• Mass action, f(S,I,A,N)=βS(I+ηA), is the easiest mathematically. This form also seems to work well for epidemics.
• Standard incidence, f(S,I,A,N)=β(I+ηA)S/N, is better suited for endemic situations or with demography present.
• More elaborate, better for fitting (if sufficient epidemic data available)

$$f\left(S,I,A,N\right)=\beta \left(I^{r}+\eta A^{s}\right)S^{p}/N^{q},$$

where r,s,p and *q* are fitting parameters.

Here, for simplicity, I use mass action incidence. With the model in each patch established, let me now turn to the metapopulation model. This is simple: indices are added to all variables and parameters to indicate what patch is being considered; movement terms are added in order to allow individuals to move between patches. For simplicity, I assume here that movement rates are independent of disease status, so that for all p,q∈P,

$$m_{pq}:=m_{Spq}=m_{Lpq}=m_{Ipq}=m_{Apq}=m_{Rpq}.$$

The rates $m_{pp}$ are defined using (2), dropping the first index because of the assumption that movement rates are independent of disease status.

The resulting |P|-SLIAR model then takes the form, for p∈P,

(4a) $$S_{p}’=-\beta_{p}S_{p}I_{p}+\sum_{q\in P}m_{pq}S_{q}$$

(4b) $$L_{p}’=\beta_{p}S_{p}I_{p}-\varepsilon_{p}L_{p}+\sum_{q\in P}m_{pq}L_{q}$$

(4c) $$I_{p}’=\left(1-\pi_{p}\right)\varepsilon_{p}L_{p}-\gamma_{Ip}I_{p}+\sum_{q\in P}m_{pq}I_{q}$$

(4d) $$A_{p}’=\pi_{p}\varepsilon_{p}L_{p}-\gamma_{Ap}A_{p}+\sum_{q\in P}m_{pq}A_{q}$$

(4e) $$R_{p}’=\gamma_{Ip}I_{p}+\gamma_{Ap}A_{p}+\sum_{q\in P}m_{pq}R_{q}.$$

Initial conditions are taken with $S_{p}\left(0\right)>0$ for all p∈P and ∃q∈P such that $I_{q}\left(0\right)+A_{q}\left(0\right)>0$ (otherwise the model is trivial); all others initial conditions are nonnegative.

### 5.2 Mathematical analysis

The focus of this paper is on running numerical simulations integrating data acquired from the Internet. However, it is always a good idea to conduct at least a local stability analysis of the model one is going to simulate, since this allows to get a sense of what the model can be expected to do. It is also useful to set what I have referred to earlier as *imputed parameters* or to get a sense of the range of values one should identify parameters in.

#### 5.2.1 Behaviour when movement is absent

Model (4) is a Kermack-McKendrick-type model, so we can expect from (Arino, Brauer, van den Driessche, Watmough, & Wu, 2007) that $I_{p}\to 0$ and $S_{p}\to S_{p\infty }$ as t→∞ for all p∈P. Let us confirm this. In patch p∈P, the model in the absence of movement is given by (3) with indices, i.e.,

(5a) $$S_{p}’=-\beta_{p}S_{p}I_{p}$$

(5b) $$L_{p}’=\beta_{p}S_{p}I_{p}-\varepsilon_{p}L_{p}$$

(5c) $$I_{p}’=\left(1-\pi_{p}\right)\varepsilon_{p}L_{p}-\gamma_{Ip}I_{p}$$

(5d) $$A_{p}’=\pi_{p}\varepsilon_{p}L_{p}-\gamma_{Ap}A_{p}$$

(5e) $$R_{p}’=\gamma_{Ip}I_{p}+\gamma_{Ap}A_{p}$$

A thorough analysis of (5) was conducted in (Arino et al., 2006), let me summarise it here. As often in epidemic models, one first seeks a disease-free equilibrium (DFE). This is obtained by setting $I_{p}=0$. Clearly, $I_{p}=0\Rightarrow L_{p}=0\Rightarrow A_{p}=0$, so the DFE has $\left(S_{p},R_{p}\right)=\left(S_{p\infty },R_{p\infty }\right)$, $\left(L_{p},I_{p},A_{p}\right)=\left(0,0,0\right)$. Also, note that $N_{p}’=\left(S_{p}+L_{p}+I_{p}+A_{p}+R_{p}\right)’=0$, which implies that $R_{p\infty }=N_{p}\left(0\right)-S_{p\infty }$. The number $S_{p}\left(0\right)-S_{p\infty }$ is the *final size* of the epidemic; it is typically expressed in the epidemiology literature in terms of the *attack rate* of the epidemic by considering the ratio $S_{p\infty }/S_{p}\left(0\right)$ (expressed as a percentage).

From (Arino et al., 2006), the *basic reproduction number* in isolated patches is given by

(6) $$R_{0}^{p}=S\left(0\right)\beta \left(\frac{1-\pi_{p}}{\gamma_{Ip}}+\frac{\pi_{p}\eta_{p}}{\gamma_{Ap}}\right).$$

This is a useful quantity to have, as it can be used to compute parameters so that $R_{0}$ is known for patches in isolation.

#### 5.2.2 Behaviour when movement is present

Let me return to the full system (5). In metapopulation models, problems are much easier to deal with when the system is written in vector form (Arino, 2017). Here, we have

(7a) S’=−β∘S∘I+MS

(7b) L’=β∘S∘I−εL+ML

(7c) $$I’=\left(I-\pi \right)\varepsilon L-\gamma_{I}I+MI$$

(7d) $$A’=\pi \varepsilon L-\gamma_{A}A+MA$$

(7e) $$R’=\gamma_{I}I+\gamma_{A}A+MR,$$

where ∘ denotes the Hadamard product. Note that the vector form is also useful when simulating the system; see Section 5.3.1. In (7), S, L, I, A, R, β, ε, $\gamma_{I}$ and $\gamma_{A}$ are vectors with |P| entries, $\pi =diag\left(\pi_{1},\ldots ,\pi_{\left|P\right|}\right)$ is diagonal, I is the identity matrix and the *movement matrix* is given by

(8) $$M=\left(\begin{array}{cccc} -\underset{p\in P\setminus \left\{1\right\}}{\sum }m_{p1} & m_{12} & \cdots & m_{1\left|P\right|}\\ m_{21} & -\underset{p\in P\setminus \left\{2\right\}}{\sum }m_{p2} & \cdots & m_{2\left|P\right|}\\ m_{\left|P\right|1} & m_{\left|P\right|2} & & -\underset{p\in P\setminus \left\{\left|P\right|\right\}}{\sum }m_{p\left|P\right|} \end{array}\right).$$

Note the negative terms on the diagonal; they are the outbound movement rates given by (2). Properties of (8) are important in the analysis of metapopulation models. Refer to (Arino, Bajeux, & Kirkland, 2019) for a list of these properties.

Without going into details here, working with a large system of ordinary differential equations such as (7) is not much different from working with the system in a single patch (5). As we did there, we start by looking for the DFE. Set I=0. Then since (I−π)ε is invertible (we have assumed that $\pi_{p}\in \left(0,1\right)$ for all p∈P), L=0. Substituting this into (7d) gives in turn A=0.

So the DFE satisfies L=I=A=0 and

MS=MR=0.

M is clearly a singular matrix, since all its columns sum to zero. This implies that MS=MR=0 have nonzero solutions $S^{⋆}$ and $R^{⋆}$. Also note that, summing equations in (7), we find N’=MN. This means that the total population in the system, $1^{T}N=\langle 1,N\rangle$, is constant, since

$$\frac{d}{dt}\langle 1,N\rangle =\langle 1,\frac{d}{dt}N\rangle =\langle 1,0\rangle =0,$$

where $1^{T}=\left(1,\ldots ,1\right)$. As a consequence, we can proceed as in (Arino & van den Driessche, 2003) and use Cramer’s rule to solve the augmented system

(9) $$\left(\begin{array}{c} 1\\ M \end{array}\right)N^{⋆}=\left(\begin{array}{c} N\left(0\right)\\ 0 \end{array}\right).$$

A sufficient condition for $N^{⋆}\gg 0$, i.e., to be entry-wise positive, is that M be irreducible, that is, that the graph of patches be strongly connected. We make this assumption and thus know there exists a unique $N^{⋆}$ that solves the system (9). Note that this solution is a function of N(0) and as a consequence, so are $S^{⋆}+R^{⋆}=N^{⋆}$. It is probably feasible to use the method in (Arino et al., 2007) to compute these distributions more precisely, but this is beyond the scope of the present work.

### 5.3 Computational analysis

#### 5.3.1 Define the vector field

Here, I adapt the code in (Arino, 2017) to the SLIAR case. As is customary when solving ODEs numerically, I first need to define the vector field. This is done in the following function. Note that I have defined a set of indices for the different epidemic stages, in order to quickly look-up the corresponding entries in the state variables vector x. This is not technically required but makes the vector field easier to read.

**Figure:**
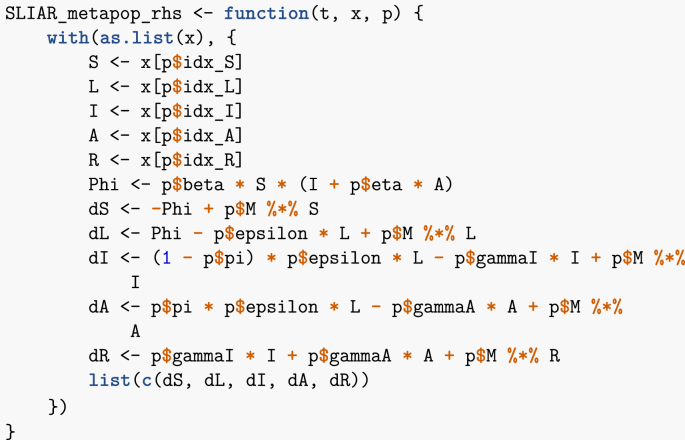

Recall that in R, * denotes the Hadamard (entry-wise) product of vectors, which I denoted ∘ in (7), while %*% denotes the usual matrix product. Thus, by writing the system in vector form, numerical integration is not much more complicated that if I were considering a single-population equivalent.

#### 5.3.2 Setting up parameters

This first example is based on the one given in (Arino, 2017). I consider five countries: Canada, China, India, Pakistan and the Philippines. The total populations of these countries are known; for instance, I obtain below the most up to date estimates from the World Bank. Also known through other means is the average number of air passengers travelling between each of these countries on a given day (estimates are from 2015).

**Figure:**
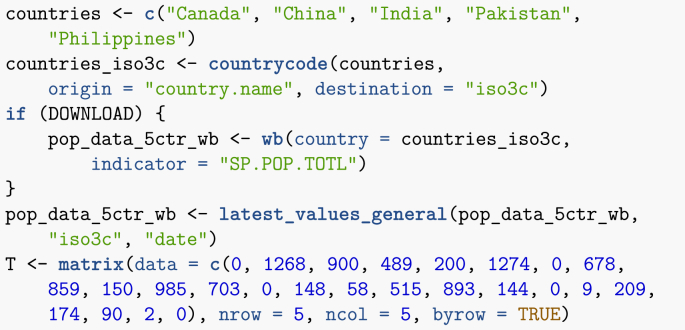

The function latest_values_general used above is part of the file useful_functions.R and is used to keep only the most recent value in a data frame. I now need to set up the movement matrix M. Here, I proceed in one of two ways that will be presented in these lecture notes, which is based on a method explained in (Arino & Portet, 2015). Suppose *X* and *Y* are two locations connected by mobility, with the population of *X* given by $N_{X}$. We seek the *per capita* rate $m_{YX}$ of movement from *X* to *Y*. If one considers a short enough time interval, then one can assume that the rate of change of the population of *X* due to travel to *Y* is governed by $N_{X}’=-m_{YX}N_{X}$. Integrating this simple linear differential equation and solving for $m_{YX}$ when t=1 gives

(10) $$m_{Y\;X}=-\text{ln}\left(1-\frac{T_{Y\;X}}{N_{X}}\right),$$

where $T_{YX}$ is the number of individuals having moved from *X* to *Y* in 1 time unit.

**Figure:**
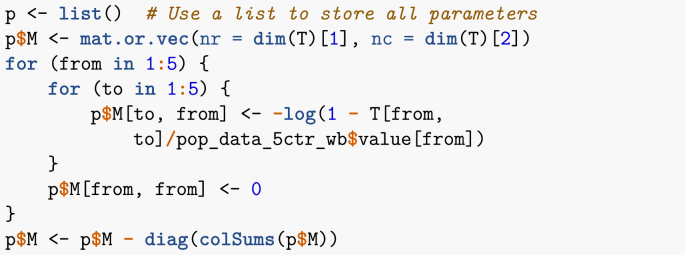

I now set up the remainder of the parameters. As indicated earlier, it is useful to keep track of indices of the different compartments.

**Figure:**
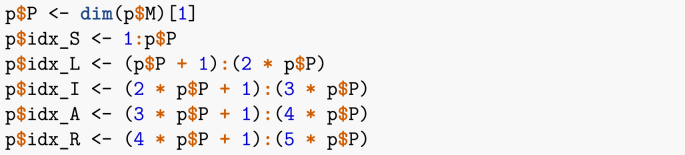

Disease-related parameters are then set. Note that values are here chosen arbitrarily, just for illustration. They are meant to roughly mimic parameters that would be used for influenza.

**Figure:**
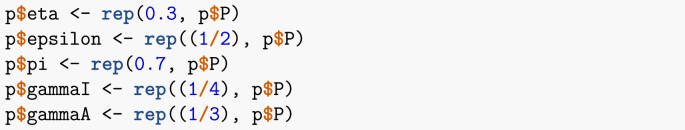

I now set up the initial conditions. As an example, suppose that there are initially two infected individuals in Canada.

**Figure:**
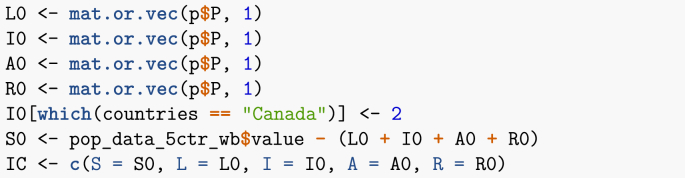

Although parameters are chosen arbitrarily, to avoid numerical issues it is useful to have some control over parameter values. Here, for instance, I set up the contact parameter β in such a way as to avoid blow up of solutions. Solving $R_{0}^{p}$ for patches in isolation as given by (6) in terms β, I obtain

$$\beta =\frac{R_{0}^{p}}{S_{p}\left(0\right)}{\left(\frac{1-\pi_{p}}{\gamma_{Ip}}+\frac{\pi_{p}\eta_{p}}{\gamma_{Ap}}\right)}^{-1}.$$

Suppose for instance that $R_{0}=1.5$ for patches in isolation; this is incorporated into the model by assuming that.

**Figure:**
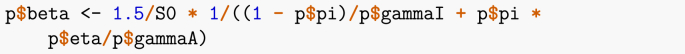

The final step is to set up the time span of the simulation, one year here.

**Figure:**
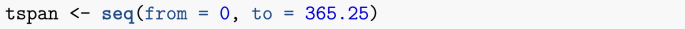

One important remark concerning the vector of times is that it can be tailored to match existing data points. I will return to this with the next example in Section 6. Finally, I call the solver,

**Figure:**
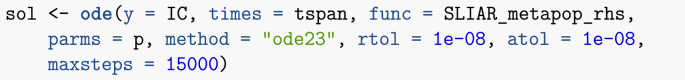

and put the result in a form that is easier to use. Note that in order to overcome numerical issues, I have changed the method used to solve the system from the default lsoda to ode23, as well as changed tolerances and the maximum number of steps. The issues arising here are linked to the difference in orders of magnitudes of the different quantities involved. For instance, population counts are in millions, whereas values of $\beta_{p}$ so that $R_{0}^{p}$ equals 1.5 are of the order of $10^{-8}$.

**Figure:**
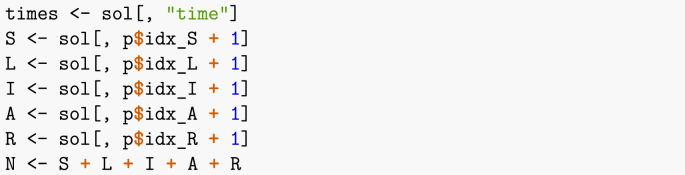

In the code above, I have to shift indices of the positions of the different variables by 1, since the first column in the result matrix contains time. This is different from the vector field itself, where x is the vector of state variables only.

**Fig. 13:**
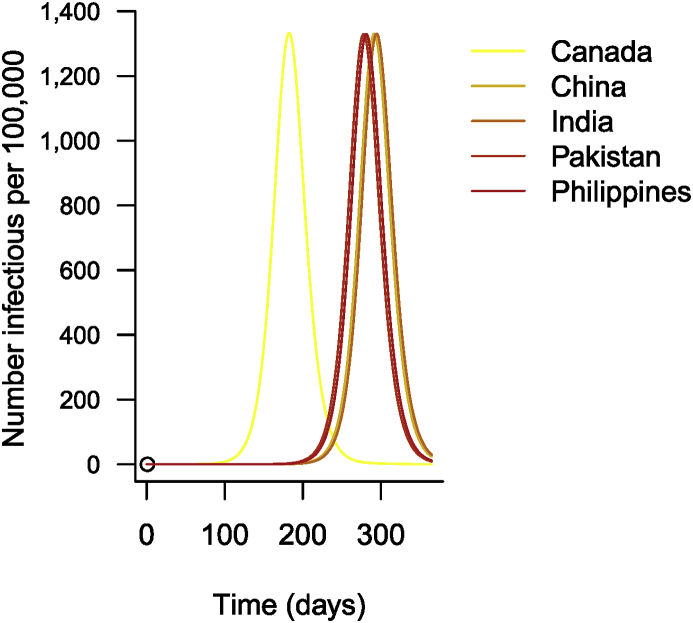
Time evolution of the number of cases of influenza in 5 countries.

Just for illustration, Fig. 13 shows the results. As is often the case with epidemiological data, I show results per 100,000 people rather than actual numbers. (Code chunk not shown.)

## 6 Spread of influenza between regions in a country

In Section 5, I considered the spread of influenza between five countries. I had air travel mobility numbers and could assume that, to a large extent, the numbers represented a large fraction of the actual means of movement, due to the distances between most of the countries involved. I did not, however, have access to precise epidemiological surveillance of influenza for all these countries and simply simulated the spread.

Here, I consider a somewhat converse problem, where the disease epidemiology is much better known, but mobility patterns need to be inferred. Staying with influenza, I look at its spread between regions of metropolitan France. Influenza data is obtained from the Réseau Sentinelles, which has been conducting practitioner-based surveillance of influenza since 1984 (Valleron et al., 1986). Also known is a rough measure of vaccine uptake in each region; the model will be (slightly) adapted to incorporate this vaccination information.

Influenza data is available online by regions in metropolitan France. Regions in France were redefined in 2016. Until then, there were 22 regions in metropolitan France (Corsica being considered part of metropolitan France). In 2016, some regions were aggregated and the number was reduced to 13. Data can be downloaded in terms of the new regions, or in terms of past regions until 2016 and of new regions since. This illustrates a common problem when dealing with data: adapting the new data to the past regions would require to infer how the data was processed. As a consequence, I use the new definition of regions.

Also available is the regional uptake of influenza vaccine. Of course, the population of regions and their geography is readily available. I now download all of the required data.

**Figure:**
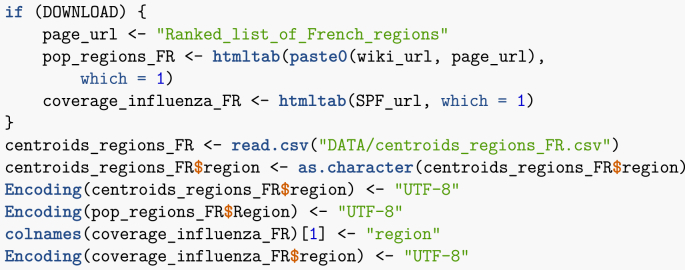

Note that here, I had to impose encoding of the downloaded data because of accents in French text. Interestingly, this issue does not arise under Linux but does in Windows.

### 6.1 Setting up a gravity matrix

In the 5 countries example of Section 5, I had access to population mobility data that, because of the distances between some of the countries, can be assumed to be a relatively good approximation of the actual travel volume. Another example where data sums up most of the actual mobility is the work in (Arino & Portet, 2015), where estimates of the number of travellers, by road this time, between a large city and a few neighbouring smaller ones are obtained and used. In the present case, though, there are two levels of complication.

1. Mobility in a country like France is multi-modal, with the main modes of mobility being air, rail and road travel.
2. Even if numbers were available for all travel modalities, they would be hard to combine.

Given this complexity, in order to approximate the volume of travel between locations using non-proprietary data, I compute a *gravity matrix*. The idea is to use the analogy of gravitation. The gravitational force $F_{ij}$ between two bodies with masses $m_{i}$ and $m_{j}$ and centres of mass *r* distance units apart is given by

$$F_{ij}=G\frac{m_{i}m_{j}}{r^{2}},$$

where *G* is the gravitational constant. Here, I use this principle with centroids (centres of gravity) of regions as centres of mass and the population of regions as their mass. The result is a symmetric matrix with zeros on the main diagonal and the gravitational constant *G* as a scalar factor. That constant is later used to set values in the movement matrix so that movement rates are commensurate with the problem under consideration.

As has become the *leitmotiv* throughout this paper, first I create a data frame with all relevant information. As region centroids are given in (degrees, minutes,seconds), they must be transformed to decimal degrees.

**Figure:**
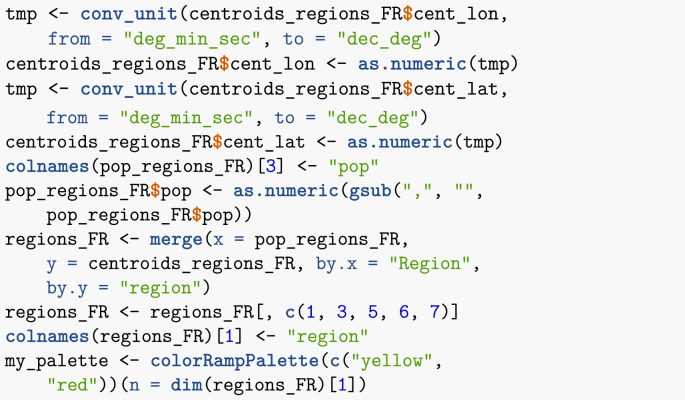

I can now compute the distances between region centroids.

**Figure:**
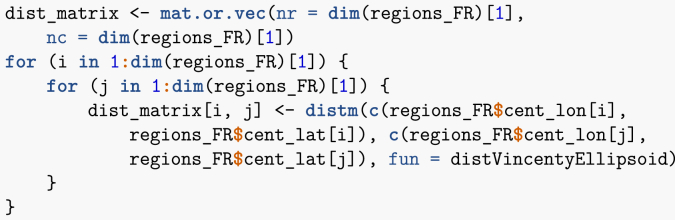

Note that I could also have used some internal R functions, such as dist, to compute the distances. Once this has been done, I compute the gravity between all pairs of regions. Note that if there were many more pairs to deal with, exploiting the symmetry of the gravity matrix would allow to gain some time.

**Figure:**
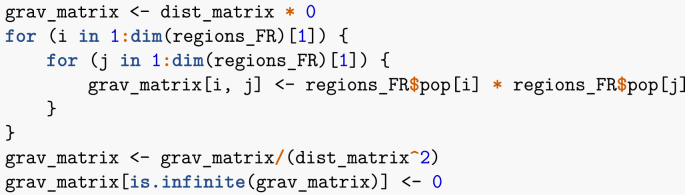

Note that the last instruction is used because in the computation of distances, I did not exclude diagonal entries and thus am dividing by zero along the diagonal. So, finally, I have the gravity matrix, where region names are abbreviated for convenience.

**Figure:**
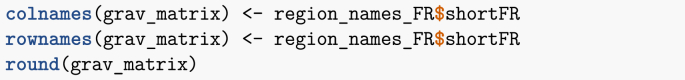

**Table:** 

	ARA	BFC	Br	Ce	Co	GE	HdF	IdF	No	Na	Oc	PL	PACA
ARA	0	604	65	213	10	335	181	640	97	402	598	137	894
BFC	604	0	27	129	2	525	148	605	57	94	83	58	99
Br	65	27	0	71	1	47	96	251	190	120	48	428	24
Ce	213	129	71	0	1	138	187	1395	183	197	84	269	48
Co	10	2	1	1	0	3	2	5	1	3	5	1	16
GE	335	525	47	138	3	0	531	1281	112	103	85	84	100
HdF	181	148	96	187	2	531	0	3652	434	112	72	153	59
IdF	640	605	251	1395	5	1281	3652	0	1225	392	227	564	172
No	97	57	190	183	1	112	434	1225	0	104	50	326	31
Na	402	94	120	197	3	103	112	392	104	0	673	314	127
Oc	598	83	48	84	5	85	72	227	50	673	0	95,292	
PL	137	58	428	269	1	84	153	564	326	314	95	0	43
PACA	894	99	24	48	16	100	59	172	31	127	292	43	0

Interestingly, the method returns realistic results: the fifth entry in the matrix is Corsica, which has a small population and is quite isolated, being an island. The unadjusted values here are too large and will be scaled down using *G* prior to being used in the numerical simulations.

### 6.2 Setting up initial conditions – vaccination coverage

I could, as I did in Section 5, use as initial conditions the population $N_{p}\left(0\right)$ in each region p∈P. Here, however, I want to incorporate the effect of vaccination. There are more elaborate methods to model vaccination, but a minimalist approach consists in assuming that vaccination simply reduces the susceptible population. Thus, I consider the initial susceptible population ${\tilde{N}}_{p}\left(0\right)=\left(1-v_{p}\right)N_{p}\left(0\right)$, where $v_{p}$ is fraction vaccinated in region p∈P. Note that this means that I am assuming that the vaccine is 100% efficacious; that is far from true, but to keep the problem simple, I make this assumption.

In our recurrent theme, I need to process vaccination data a little in order to be able to use it in simulations. (Chunk not shown.)

Setting initial conditions then proceeds as in Section 5, except that the total population $N_{p}\left(0\right)$ reflects the percentage vaccinated. I start with several cases in, say, Île-de-France (the region comprising Paris). (Chunk not shown.)

For the movement matrix M, I start with the gravity matrix I already computed, which I also store for future use. Here, I normalise by imposing that movement rates in the matrix should not be larger than 0.005, a somewhat arbitrary value I have often used in metapopulation models and gives usually reasonable results in terms of population movement.

**Figure:**
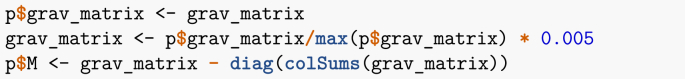

If information about mobility volumes is available, one can “invert” the method used in Section 5 in order to obtain finer estimates for the value of *G*.

**Fig. 14:**
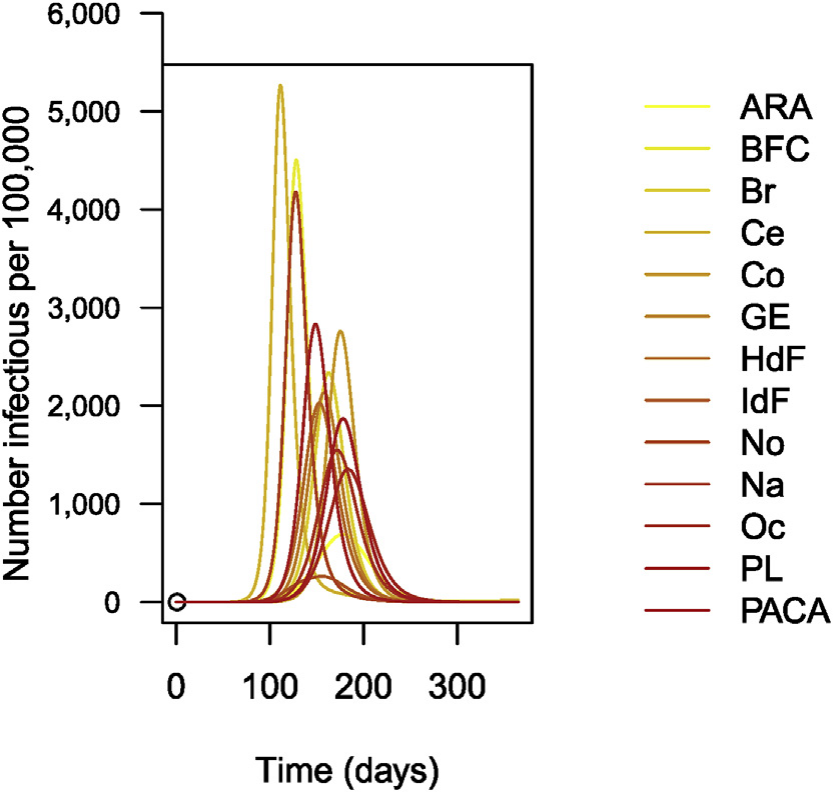
Number of cases of influenza per 100,000 inhabitants (simulation results).

The following is then done (chunks not shown). Parameters are set as before. As I did earlier, I suppose that $R_{0}=1.5$ for patches in isolation. This having been done, I carry out the numerical integration and plot the solution, giving Fig. 14.

### 6.3 Using epidemiological data

Let me load the Réseau Sentinelles data and prepare it for use. Note that in the case Réseau Sentinelles, where data typically changes weekly and the equipment does not necessarily support very frequent queries, it is good practice to keep a local cache of the data and refresh only in case of change. So, when loading the data, I use a function called nice_load (found in the useful_functions.R electronic appendix) that handles this. Some more elaborate mechanisms are available, but I felt it was worth illustrating how to do this here. The data has dates in the form YYYY-WW, where WW is the week number. I need to transform this into a regular date.

**Figure:**
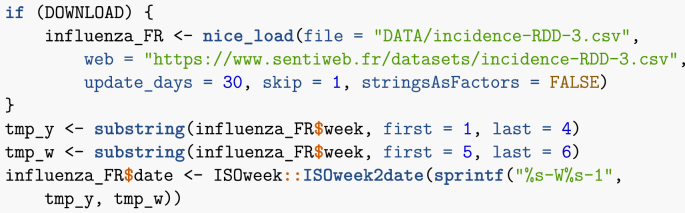

It is always a good idea to explore the data. At the very least, it should be plotted in order to get a sense of the general behaviour. Let me create a few variables that allow to access the data easily. (Chunk not shown.)

**Fig. 15:**
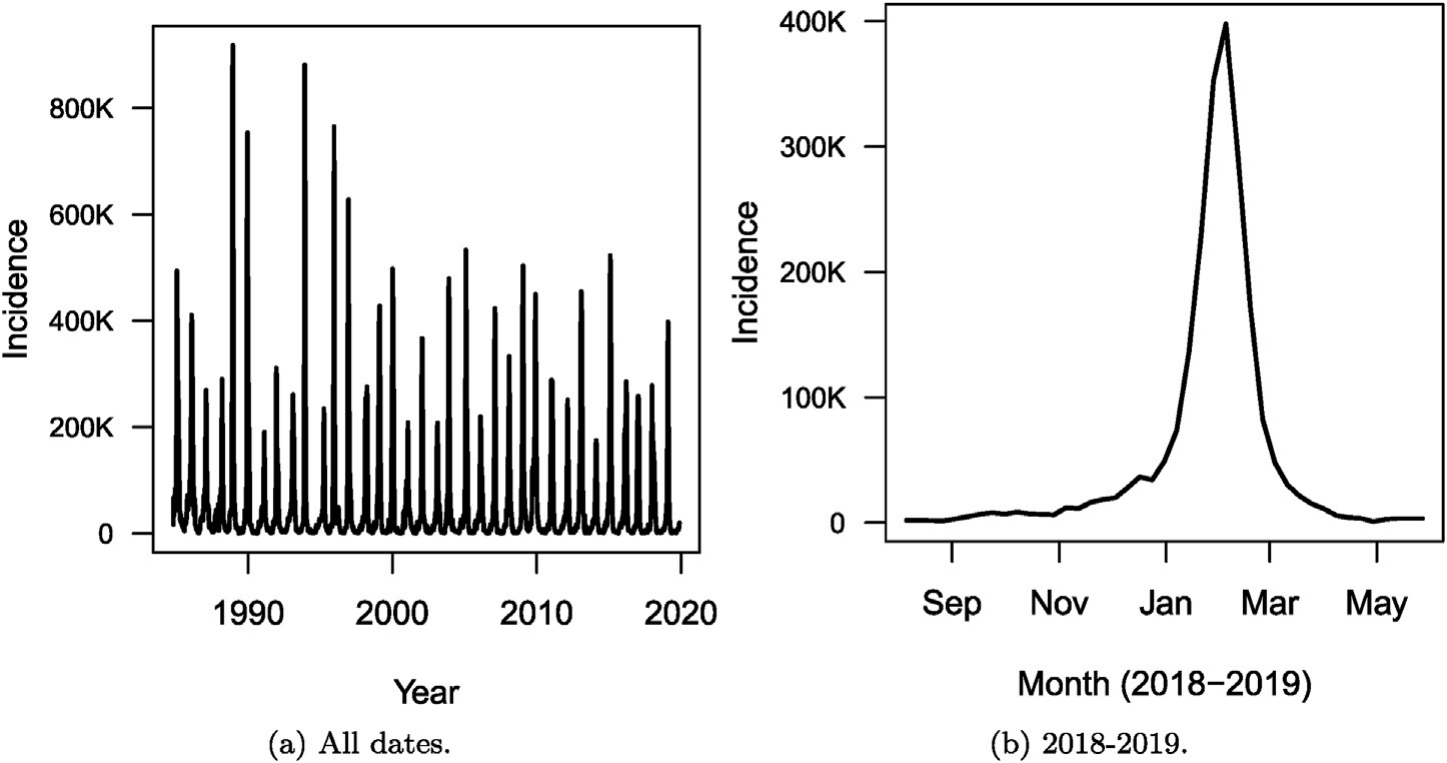
Weekly number of cases of influenza in France as reported by the Réseau Sentinelles. (a) All dates. (b) 2018–2019 epidemic season.

Let me take a look at the data. First, in Fig. 15, I show the total weekly number of cases over the entire country for the entire dataset.

Let me now select the latest influenza season at the time of writing. An end date in 2019 is specified because future uses of the code provided here would extend the plot to more than one season.

**Figure:**
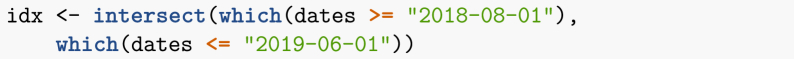

I then plot, in Fig. 15, the number of cases during the 2018–2019 epidemic season for the entire country and in Fig. 15, the breakdown of these numbers region by region (per 100,000 people, in this case, because of the wide variation of population between regions).

### 6.4 Numerical simulation of the system

**Fig. 16:**
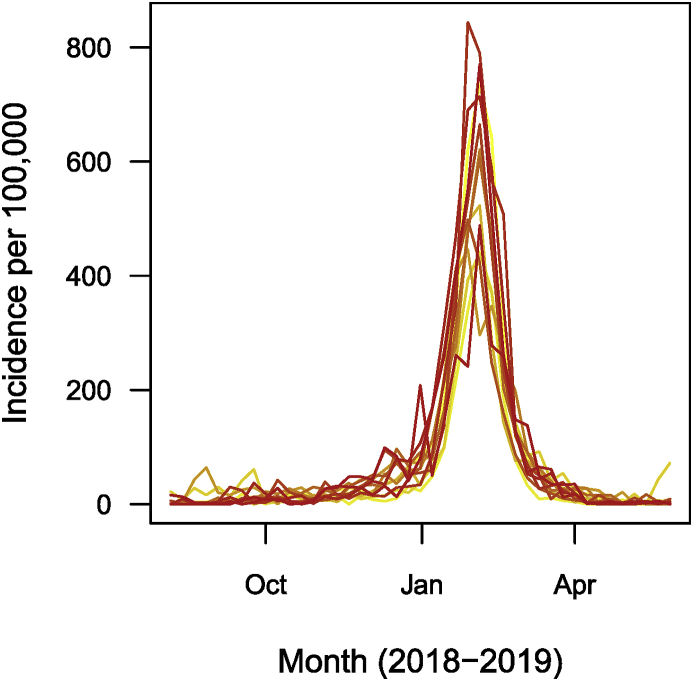
Weekly number of cases of influenza in each of the 13 regions of metropolitan France for the 2018–2019 epidemic season, as reported by the Réseau Sentinelles. Numbers are per 100,000.

For initial conditions, I use actual incidence, not the number of cases per 100,000 that I plotted in Fig. 16. For simplicity, I assume that all reported cases are symptomatically infectious. I further assume that, as in System (3), a fraction $\pi_{p}$ of cases in patch p∈P are asymptomatically infectious, so that if there are initially $I_{p}\left(0\right)$ symptomatic cases, there are also $\left(1-\pi_{p}\right)I_{p}\left(0\right)$ asymptomatic ones.

**Figure:**
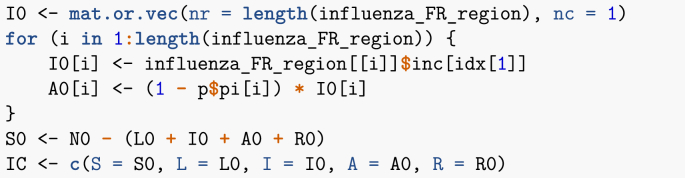

First, let me run a naïve simulation. Note that for time span and time points, I use the dates I selected. This feature, that was mentioned earlier, is useful when minimizing errors.

**Figure:**
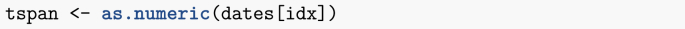

**Fig. 17:**
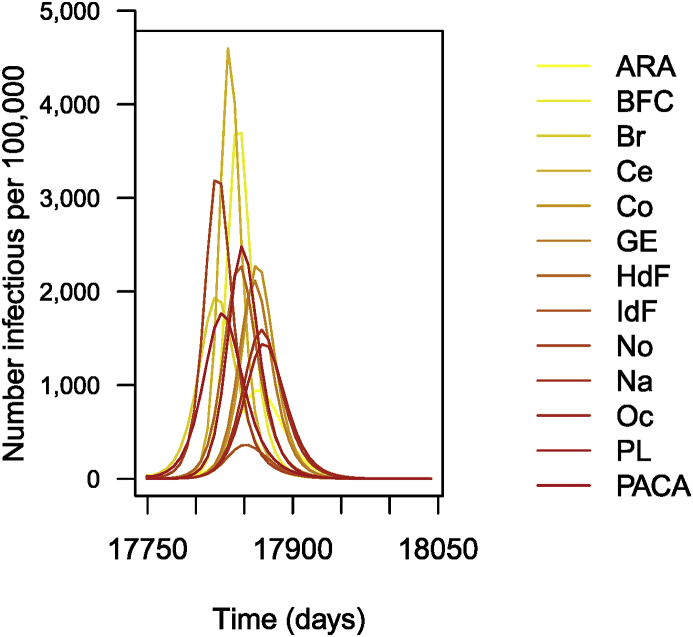
Simulation of 1 year of influenza propagation in France. Numbers are per 100,000.

The remainder of the call (chunk not shown) then proceeds as previous instances; the result is shown in Fig. 17.

Let me now carry out a naïve parameter identification routine. As mentioned in the Introduction, the procedure carried out here is very simple and much more information will be gained by following the techniques found in lecture notes from other authors. The results of the procedure are not good by any means and much more attention should be paid to the details in actual work.

The way I proceed is to allow two parameter types to vary: the individual value of $\beta_{p}$ in the patches and the gravity constant *G*. First, I need a function similar to the one used in Section 3.1.2, which, given values of $\left(\beta_{1},\ldots ,\beta_{13},G\right)$, returns an error, taken here as the sum of the square of the differences between data points and the simulated solutions. The vector v passed as an argument to this function contains the parameters that change, while param is the remained of parameters.

**Figure:**
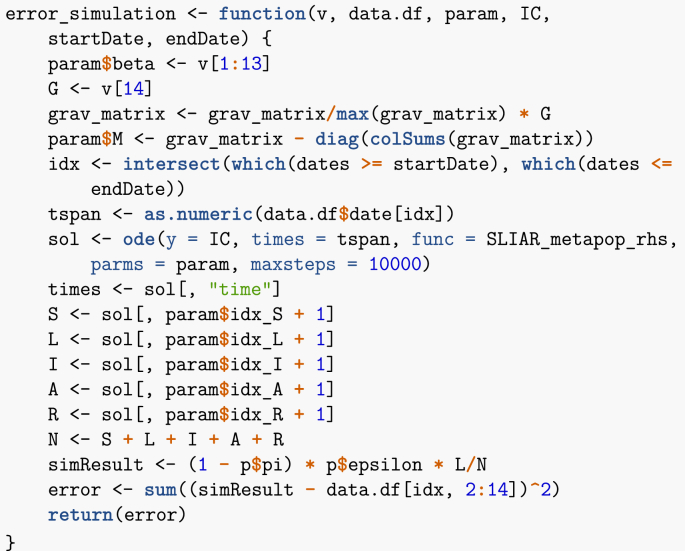

Now that this function is ready, I can use a genetic algorithm to minimise the error. Genetic algorithms work by maximising the so-called fitness function, hence I give as argument the negative of the function I just defined. Note that since the data is incidence data, I need comparable model output. Because I assume that neither latently infected nor asymptomatic cases are detected, it follows that comparison is to the rate of apparition of new symptomatically infectious cases, (1−π)εL.

**Figure:**
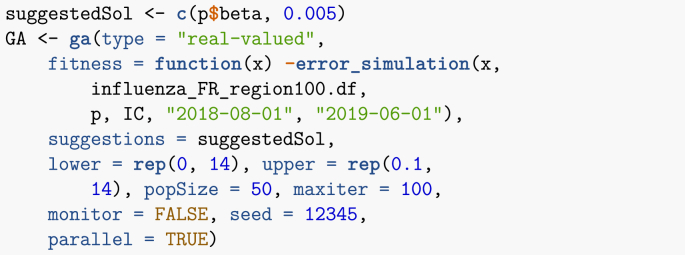

Once the method has run, results can be accessed in GA@solution. Recall that I have used parameters as $\left(\beta_{1},\ldots ,\beta_{13},G\right)$, so.

**Figure:**
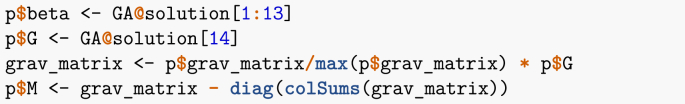

**Fig. 18:**
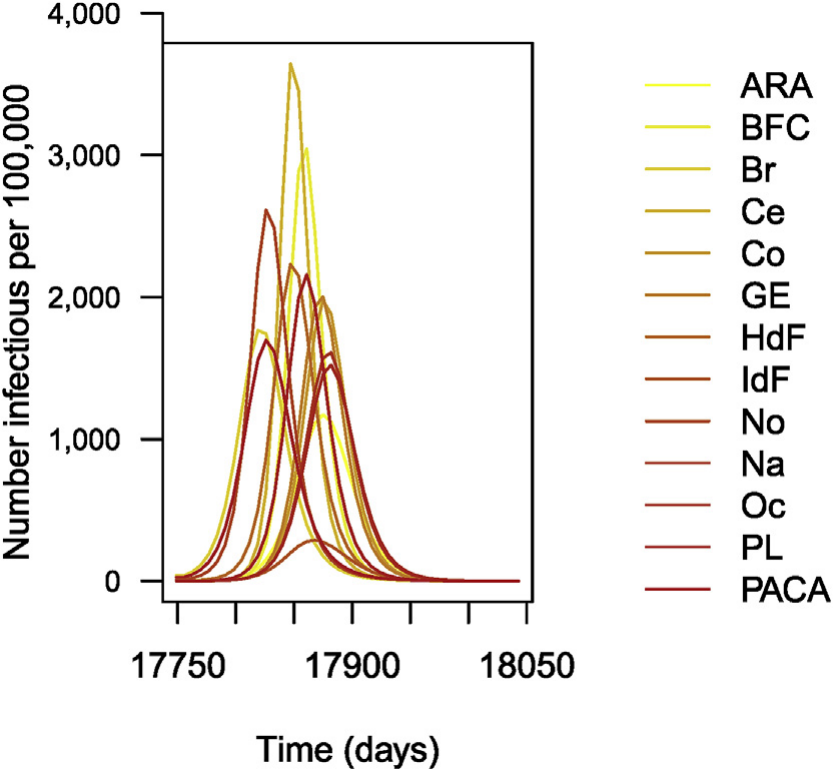
Weekly number of cases of influenza in each of the 13 regions of metropolitan France for the 2018–2019 epidemic season. Parameters obtained by genetic algorithm optimisation. Numbers are per 100,000.

Once this is done, I solve the ODE numerically and plot the result (chunk not shown). The plot is shown in Fig. 18. Obviously, the results are not fantastic. Much more could be done to obtain a better fit; I will refer to other lecture notes in this special issue for details. Two potential ways to address the limitations here are the following.

1. Switch to time units of weeks. Recall that data is the number of new cases detected in one week. The time unit in the simulation is the day, so that (1−π)εL is expressed per day. To decrease the distance between data and simulation, the genetic algorithm thus selects larger values of β, resulting in an earlier epidemic.
2. I could also play with the date of start of the simulation and make it a parameter to identify as well.

## Conclusion

To conclude, let me start by justifying in hindsight the use of the type of methods presented here, by giving a very brief overview of the path that led me toward this type of problems. My systematic use of data in epidemic models began with my work with the Bio.Diaspora Project, now the company Bluedot (link). In this context, we have a very rich database focused on human mobility; see some details in (Arino & Khan, 2014). We use these data to understand global public health risks, with emphasis on those aspects of risk linked to mobility and, in particular, air travel. Questions that arise in this context are twofold. First, if an alert is generated by a disease surveillance system, what is the potential for the event being reported on to transform into a significant event with potential for regional or international spread? Second, if the event has potential for transborder spread, what are the most likely next locations it will affect? Clearly, considering these questions requires gathering data on a scale that cannot be realistically achieved without programmatic approaches. Hence, the move towards automated or, at the very least, semi-automated information retrieval. Automated report generation, on the other hand, is prompted by the need to summarise the same *indicators* about different locations based on the location of an alert.

The methods presented here should be seen as a component in modelling work. Data can help modellers get a better sense of the context they are operating in. Data can also help modellers conduct better numerical investigation of their model. However, data is not a substitute to proper modelling work, nor does it absolve the modeller from conducting some mathematical analysis. In any case, programmatic techniques of data acquisition such as the ones I illustrate in these notes should, in my view, become part of the arsenal of techniques mathematical epidemiologists are familiar with. Ideally, mathematical epidemiologists should also familiarise themselves with the concept of *reproducible research*, which is illustrated by the medium chosen for these notes. While the model formulation and mathematical analysis part of our work is, of course, inherently reproducible, the numerics or data components are not necessarily and adopting reproducible research ideas would be sometimes helpful.
